# Intracellular Antioxidant and Anti-Inflammatory Effects and Bioactive Profiles of Coffee Cascara and Black Tea Kombucha Beverages

**DOI:** 10.3390/foods12091905

**Published:** 2023-05-06

**Authors:** Amanda L. Sales, Amaia Iriondo-DeHond, Juliana DePaula, Mafalda Ribeiro, Isabel M. P. L. V. O. Ferreira, Marco Antonio L. Miguel, María Dolores del Castillo, Adriana Farah

**Affiliations:** 1 Núcleo de Pesquisa em Café Prof. Luiz Carlos Trugo (NUPECAFÉ), Laboratório de Química e Bioatividade de Alimentos, Instituto de Nutrição, Universidade Federal do Rio de Janeiro, Avenida Carlos Chagas Filho, 373, CCS, Bl. J, Rio de Janeiro 21941-902, Brazil; 2 Laboratório de Biociencia de Alimentos, Instituto de Investigación em Ciencias de La Alimentación (CIAL) CSIC-UAM, Calle Nicolás Cabrera, 9, Campus de la Universidad Autónoma de Madrid, 28049 Madrid, Spain; 3 Departamento de Nutrición y Ciencia de los Alimentos, Sección Departamental de Nutrición y Ciencia de los Alimentos, Facultad de Veterinaria, Universidad Complutense, 28040 Madrid, Spain; 4 LAQV/REQUIMTE, Laboratório de Bromatologia e Hidrologia, Departamento de Ciências Químicas, Faculdade de Farmácia da Universidade do Porto, 4099-030 Porto, Portugal; 5 Laboratório de Microbiologia de Alimentos, Instituto de Microbiologia Paulo de Góes, Universidade Federal do Rio de Janeiro, Avenida Carlos Chagas Filho, 373, CCS, Bl. I, Rio de Janeiro21941-902, Brazil

**Keywords:** dried coffee cherry pulp, coffee cascara, coffee byproduct, fermentation, intracellular antioxidant capacity, anti-inflammatory effect, sustainability

## Abstract

Kombucha is a functional beverage obtained through fermentation of sweetened *Camellia sinensis* infusion by a symbiotic culture of bacteria and yeasts that exerts many beneficial biological effects, mostly related to its antioxidant and anti-inflammatory effects. Alternative raw materials have been used to create new kombucha or kombucha-like products. Coffee is the most important food commodity worldwide and generates large amounts of by-products during harvest and post-harvest processing. The main coffee by-product is the dried fruit skin and pulp, popularly known as cascara. To date, no studies have evaluated the potential bioactivity of coffee cascara kombucha. In this study, we aimed to measure and compare the effects of infusions and kombuchas made with arabica coffee cascaras (*n* = 2) and black tea leaves (*n* = 1), fermented for 0, 3, 6, and 9 days on the intracellular production of Reactive Oxygen Species (ROS) and Nitric Oxide (NO) in model cells. Oxidative stress was induced in HK-2 cells with indoxyl sulfate (IS) and high glucose (G). Inflammation was induced with lipopolysaccharide (LPS) in RAW 264.7 macrophage. The contents of phenolic compounds, caffeine, and other physicochemical parameters were evaluated. To the best of our knowledge, this is the first study providing information on the bioactive profile and on the potential biological effects of coffee cascara kombucha. Fermentation caused the release of bound phenolic compounds from the infusions, especially total chlorogenic acids, with an average increase from 5.4 to 10.7 mg/100 mL (98%) and 2.6–3.4 mg/100 mL (30%) in coffee cascara and black tea kombucha, respectively, up to day 9. All evaluated beverages reduced (*p* < 0.0001) similarly the intracellular ROS (41% reduction, on average) and uric acid (10–55%) concentrations in HK-2 model cells, reversing the induced oxidative stress. All beverages also reduced (*p* < 0.0001, 81–90%) NO formation in LPS-induced macrophages, exhibiting an anti-inflammatory effect. These potential health benefits may be mostly attributed to polyphenols and caffeine, whose contents were comparable in all beverages. Coffee cascara showed similar potential to *C. sinensis* to produce healthy beverages and support sustainable coffee production.

## 1. Introduction

According to epidemiological studies, *Camellia sinensis* tea consumption exerts several beneficial biological effects, such as helping prevent cardiovascular diseases, type 2 diabetes, and colorectal cancer [1,2,3,4]. These effects mostly relate to *C. sinensis* antioxidant and anti-inflammatory activity [5,6]. Kombucha tea is defined as a beverage obtained through the fermentation of sweetened green or black (*C. sinensis*) infusion by the action of a Symbiotic Culture of Bacteria and Yeast (SCOBY) [7,8,9]. In vitro and animal studies showed that *C. sinensis* biological activities remain in kombucha [10,11,12,13,14,15,16,17].

In recent years, there has been a global resurgence in the interest in fermented foods, especially in Western society, where consumption levels had decreased in previous decades. In this scenario, kombucha is an alternative to traditional soft drinks and light-alcohol drinks because of the high amount of bioactive compounds and claims against the pathogenesis of global chronic diseases [18,19]. The global kombucha market size was valued at USD 2.64 billion in 2021 and is expected to expand at an annual growth rate (CAGR) of 15.6% from 2022 to 2030 [20]. Consumer awareness and popularity are the main reasons behind the current trend of the flourishing market and active research on kombucha [21]. Along with this trend, the consumer demand for healthy flavorsome foods continuously increases. Recently, raw materials different from *C. sinensis* tea, for example, fruit or vegetable juices and cocktails, herbal or plant infusions, and food industry by-products, have been used to create new kombucha (containing *C. sinensis* tea) and kombucha-like (containing only alternative extracts) functional products [22].

Coffee is the most important food commodity worldwide and ranks second, after petroleum, among all commodities [23]. In the last 20 years, global coffee production has consistently increased [24]. According to the International Coffee Organization (ICO), approximately 10 million tons of seeds were produced worldwide in 2021/2022 [25], generating large amounts of by-products during harvest and post-harvest processing [26]. With the prospect that coffee can reach a totally sustainable environment, it should be noted that the associated by-products still have not gained enough popularity to be reused.

**Figure 1:**
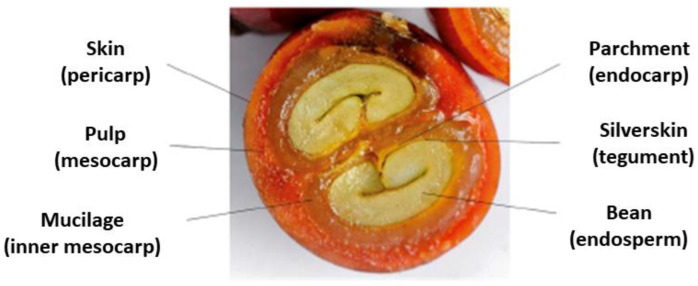
Transversal section of a ripe coffee cherry. Reference: del Castillo et al. [39], with permission from the Royal Society of Chemistry.

The main coffee by-product is the dried cherry pulp, as officially named by the European Food Safety Authority (EFSA) [27] and popularly known worldwide as coffee cascara (Figure 1). It is generated by separating coffee beans from the cherry skin and pulp [28]. The coffee pulp alone corresponds to approximately 28% of the coffee fruit on a dry weight basis, and the skin, approximately 12% [26]. These wastes are contamination sources and may lead to water and soil pollution and negative environmental impacts, given the high concentrations of caffeine, tannins, and lower molecular weight polyphenols [28,29]. On the other hand, cascara has the potential as a natural, sustainable source of bioactive compounds such as chlorogenic acids, flavonoids, and caffeine, soluble fibers, and micronutrients such as ascorbic acid and minerals [30,31,32,33,34]. Indeed, several potentially beneficial effects of coffee cascara as a whole product, such as antioxidant, anti-inflammatory, antibacterial, adipogenic, and lipolytic effects, have been observed in vitro [35,36,37,38].

In Europe, coffee cascara was considered a novel food by the European Food Safety Authority (EFSA) from 2015 until 2021, when it was authorized to be used in the European market [40] as a safe food ingredient for human consumption, with multifunctional properties. It can be used in bread production as flour [31], added to food matrices like yogurts [41], or simply for infusion preparation [26,27,36]. The fermentation of fresh coffee pulp and cascara infusion has been recently proposed [42,43,44]. However, to our knowledge, there are no reports evaluating the bioactive profile and the potential biological effects of coffee cascara kombucha. This study aimed to evaluate the potential antioxidant and anti-inflammatory effects of infusions and kombuchas prepared with coffee cascara tea during fermentation, compared with plain black tea infusion and kombuchas. For this, we used human-induced proximal tubular (HK-2) and murine macrophage (RAW 264.7) cell models. Additionally, we provided information on the bioactive compounds of coffee cascara kombucha.

## 2. Materials and Methods

### 2.1. Reagents

Indoxyl sulfate (IS), uric acid (UA), glucose (G), tert-butyl hydroperoxide (tBOOH), dimethyl sulfoxide (DMSO), 3-(4,5-dimethylthiazole-y)-2,5-diphenyltetrazolium bromide (MTT) and 21,71-dichlorodihydro-fluorescein diacetate (DCFH-DA), sodium nitrite, lipopolysaccharide from *E.coli* O55:B5 (LPS) were purchased from Sigma Chemical (Sigma-Aldrich, St. Louis, MO, USA). Dulbecco’s Modified Eagle’s Medium (DMEM) was purchased from Lonza (Basel, Switzerland). L-glutamine, antibiotics (penicillin and streptomycin), and trypsin were obtained from Gibco (Invitrogen Co., Grand Island, NY, USA), and fetal bovine serum (FBS) was obtained from Hyclone (GE Healthcare, Chicago, IL, USA). Catechins, including (-)-catechin (>98%), (-)-epicatechin (>98%), (-)-gallocatechin (>98%), (-)-epigallocatechin, (-)-catechin gallate (>98%), (-)-gallocatechin gallate (>98%), and (-)-epigallocatechin gallate (>98%), gallic acid (≥99%), 5-caffeoylquinic acid (≥95%) rutin (hydrate,≥94%), quercetin (hydrate, ≥95%), kaempferol (≥97%), caffeic acid (≥98%), ferulic acid (≥99%), p-coumaric acid (≥98%), sinapic acid (≥98%), benzoic acid (≥99.5%), 3,4-dihydroxybenzoic acid (≥97%), hippuric acid (≥98%), 3,4-dihydroxyphenylacetic acid (≥98%), 4-hydroxyphenylacetic acid (≥98%), vanillic acid (≥97%), dihydrocaffeic acid (≥98%) and caffeine (≥99%) for HPLC were provided by Sigma Chemical Co. For dicaffeoylquinic acids (diCQA), a mixture of 3,4-diCQA; 3,5-diCQA; and 4,5-diCQA from Carl Roth (Karlsruhe, Germany) was used. Feruloylquinic acids (FQA) were synthesized from 3-feruloylquinide and 4-feruloylquinide (FQL) by hydrolysis in 50% aqueous tetrahydrofuran [45]. Sucrose was provided by PROQUIMIOS Produtos Científicos, Rio de Janeiro, Brazil; glucose and fructose were provided by VETEC Química Fina, Rio de Janeiro, Brazil.

### 2.2. Raw Materials

A leading commercial black tea brand was purchased in a Rio de Janeiro food market; organic arabica coffee (Coffea arabica) cascara samples were acquired from producers (dry processed CCB from Espírito Santo, Brazil and wet processed CCN from Nicaragua).

### 2.3. Infusions Preparation, Kombucha Consortium, and Fermentation

The black tea (BT) and coffee cascara (CC) infusions were prepared at 3% (weight/volume—*w*/*v*), pouring water at 95 °C over the raw material, letting it steep for 10 min, and filtering the mixture using a regular paper filter (Mervilab S.A., Madrid, Spain) for bulk tea.

- Kombucha Consortium and fermentation: The Kombucha Consortium was part of the collection of the Microbiology Institute of the Federal University of Rio de Janeiro in Brazil. Previously cultivated in green tea, the consortium was separately fermented 3 times in black tea and in coffee cascara tea infusion prior to experimental use in order to stabilize the microbial consortium in these matrixes [46]. All kombucha beverages were prepared according to the protocol described by Nummer [47];
- Black tea kombucha (BT K): Black tea kombucha was prepared by mixing 10% (volume/volume—*v*/*v*) of black tea starter, 80% black tea infusion (weight/volume—*w*/*v*), 10% (*w*/*v*) sugar, and 2.5% (*v*/*v*) of a Symbiotic Culture of Bacteria and Yeast (SCOBY) and letting the mixture ferment for 14 days at 23 °C (Sanyo™ MIR-154PE, Sanyo Electric Co., Ltd., Osaka, Japan). Samples were collected before fermentation (day 0) and after 3, 6, and 9 days of fermentation;
- Coffee cascara kombucha (CCB K and CCN K): Coffee cascara kombuchas were prepared using 80% (*v*/*v*) of the coffee cascara infusion, 10% (*v*/*v*) of the black tea kombucha, 10% (*w*/*v*) sugar, and 2.5% (*w*/*v*) of SCOBY. The mixture was allowed to ferment at 23 °C (Sanyo™ MIR-154PE, Sanyo Electric Co., Ltd., Japan). Samples were collected before fermentation (day 0) and after 3, 6, and 9 days of fermentation.

All beverages were sterilized with a 0.22 µm membrane before chemical analyses and biological effect evaluation. The beverages were diluted ten times prior to the cell experiments (see Section 2.8 and Section 2.9). 

### 2.4. DNA Extraction, Amplicon Sequencing Data Analysis and Library Preparation

DNA was extracted from the liquid and the biofilm samples after 14 days of fermentation (starter culture) for black tea kombucha and after 9 days of fermentation for all other beverages, following the protocol described by Yamanaka et al. [48]. 

The identification of bacteria (16S rRNA gene) and yeasts (ITS1 region) was performed by using a high-performance DNA sequencing method using a MiSeq Sequencing System (Illumina Inc., San Diego, CA, USA). The library preparation and DNA sequencing followed the protocol of Neoprospecta Microbiome Technologies, Brazil. Amplification with primers to the V3–V4 region of the rRNA 16S gene (341F-CCTACGGGRSGCAGCAG and 806R-GGACTACHVGGGTWTCTAAT, and the ITS region (ITS1-GAACCWGCGGARGGATCA and ITS2-GCTGCGTTCTTCATCGATGC) was performed for bacteria and yeasts, respectively. The sequences were analyzed by pipeline Sentinel. In pipeline Sentinel, the archives fastQ were evaluated for quality Phred (QP) using the software FastQC v.0.11.8 [49]. Analyses were performed in triplicate.

Clusters with an abundance lower than 5 were removed according to the methodology proposed by Smyth et al. [50]. The taxonomic identifications were carried out with BLASTN v.2.6.0+ [51]. As for the species definition, within the 20 hits returned for each cluster, a Python instruction evaluated whether 1 of the 3 requirements would be met by the hits: (1) highest bit-score; (2) lowest e-value; and (3) taxonomies with greater representation. The hits that fit 1 of the previous items were chosen as representative species. The bacteria and yeast species were defined using 99% and 97% of identity, respectively.

### 2.5. Analysis of Titratable Acidity, pH, Soluble Solids, and Sugars

Total soluble solids were evaluated using a handheld refractometer (Pocket Refractometer Pal-1, ATAGO, Tokyo, Japan). Results were expressed in °Brix. The total titratable acidity was determined by titration with 0.1 N NaOH and phenolphthalein as an indicator, according to Adolfo Lutz Institute [52]. Results were expressed in mEq/L. pH was measured using a pH meter (Kasvi K39-0014PA, São José dos Pinhais, Paraná, Brazil).

Sucrose was analyzed according to Wischral et al. [53], using a High-Performance Liquid Chromatography-Refractive Index Detector (HPLC-RID)system (mod.# 2414, Waters, Milford, MA, USA), using a Hi-Plex column H 8 μm (300 × 7.7 mm; Agilent, Santa Clara, CA, USA) at 30 °C with 20 μL of injection volume and H_2_SO_4_0.005 mol/L as mobile phase at 0.4 mL/min. For glucose and fructose, the column temperature was 60 °C, and the mobile phase flow was 0.6 mL/min. External standards were used for sugar identification and quantification.

### 2.6. Analysis of Bioactive Compounds

#### 2.6.1. Analysis of Catechins, Chlorogenic Acids and Free Phenolic Acids

The analyses of catechins were carried out according to Liang et al. [54], using a High-Performance Liquid Chromatography-Diode Array Detector (HPLC-DAD) system composed of 2Jasco PU-2080 HPLC pumps, a column heater—Model 7981—Jones Chromatography, an MD-2010 Plus multi-wavelength diode array detector and a Jasco As-950 intelligent sampler. The column was a reversed-phase Waters Spherisorb^®^ C18 5 µm ODS2. The Borwin PDA Controller Software (JMBS Developments, Le Fontanil, France) was used. Gradient elution was performed with a mixture of 2solvents containing acetonitrile/acetic acid/water at 1 mL/min. Detection was performed at 280 nm. An external standard curve containing 7 catechins (catechin, catechin gallate, epicatechin, gallocatechin gallate, epigallocatechin gallate, epigallocatechin and gallocatechin) was prepared in de-ionized water for calibration and quantification of catechins.

The analyses of 9 chlorogenic acids (3 caffeoylquinic acids, 3 feruloylquinic acids, and 3 dicaffeoylquinic acids) and free phenolic acids were performed using a HPLC-DAD system, according to Farah et al. [55] and Duarte and Farah [56], with adaptations, using a reverse-phase column (Magic C30, 150 × 2 mm × 5 μm, 100 Å, Michrom Bioresources Inc., Auburn, CA, USA). The 2-phase LC mobile system consisted of 0.3% formic acid (eluent A) and methanol (eluent B). The gradient was programmed to operate with a flow rate of 1.0 mL/min, and DAD was set at 325 nm for chlorogenic acids and 280 nm for phenolic acids. Identification and quantification were performed using external standard curves and molar extinction coefficients. LC-MS and UV spectra were used to confirm the peaks’ identities.

#### 2.6.2. Analysis of Caffeine

The analyses of caffeine were performed using an HPLC-DAD system set to 272 nm, according to Farah et al. [57] with adaptations, using a reverse-phase column (Magic C30, 150 × 2 mm × 5 μm, 100 Å, Michrom Bioresources Inc., Auburn, CA, USA) and 40% methanol as mobile phase, running at 1.0 mL/min.

### 2.7. Cell Viability Assay

The effect of the test beverages on cell viability was measured using the MTT assay [58]. HK-2 and RAW 264.7 cells were cultured at a density of 1.0 × 10^4^ and 8.0 × 10^4^ cells per well of a 96-well plate, respectively. After 24 h culture (at 37 °C, 5% CO_2_, in a humidified incubator—BINDER CB series 2010, Tuttlingen, Germany), HK-2 cells were treated with the beverages diluted in DMEM culture medium without FBS (beverages with IS at 2.5 mM and glucose at 25 mM were diluted in PBS for 3 h). RAW 264.7 cells were also treated with the beverages diluted in DMEM culture medium without FBS but with 1 µg/mL LPS. DMSO (50%) was used as death control. Subsequently, cells were incubated in MTT solution (0.5 mg/mL) for 1 h at 37 °C and 5%CO_2_. The supernatant was removed, 100 μL of DMSO was added, and the optical density at 570 nm was measured using a microplate reader (BioTek Synergy HT Multi-Mode Microplate Reader, Winooski, VT, USA). Experiments were carried out 3 times in triplicate.

### 2.8. Indoxyl Sulfate-Induced Oxidative Stress in HK-2 Diabetic Nephropathy Cell Model

To evaluate the potential antioxidant capacity of the studied beverages, we induced the diabetic nephropathy stress condition and production of Reactive Oxygen Species (ROS) in HK-2 cells using IS and high G solution. The analysis was performed by measuring the fluorescence intensity of the DCFH-DA probe, which was proportional to the amount of ROS formed [59,60].

Human kidney proximal cell line HK-2 cells (ATCC, Manassas, VA, USA, accession # CRL-2190) were part of the collection of Instituto de Investigaciones Biomédicas Alberto Sols (Madrid, Spain). The cells were cultured in Dulbecco’s Modified Eagle’s Medium (DMEM), with 1.0 g of glucose and supplemented with 10% fetal bovine serum (FBS), 1% L-glutamine and 1% penicillin/streptomycin. Following this, they were incubated in a 96-well plate at a density of 1.0 × 10^4^ cells per well for 24 h at 37 °C and 5% CO_2_ in a humidified incubator. A solution containing IS 2.5 mM and G 25 mM was prepared in phosphate buffer saline (PBS) and sterilized with a membrane with 0.22 µm pore before the experiments. The cells were then treated with 100 μL of the following solution: test beverage (100 µL), 25 μL of IS solution and 25 µL of G solution, and 850 µL DMEM culture medium without FBS. Then, they were incubated for 3 h. 100 μL of the aforementioned solution containing PBS instead of the test beverage was used as a control for the nephropathic oxidative stress condition.

A 10 mM solution of DCFH-DA probe was prepared (5 mg in 1 mL DMSO). Then, 800 μL of DMSO was added to 50 μL of this solution. After 3 h of beverage extract incubation, cells were pre-loaded with 2.5 μL/well of this last solution for 30 min at 37 °C and 5% CO_2_. After incubation, DCFH becomes dichlorofluorescein (DCF) due to intracellular oxidants and will emit fluorescence. Next, the culture medium was removed, and cells were washed with 100 μL phosphate-buffered saline (PBS). The cells were then treated again with 100 μL of the same test beverages with IS and G. tBOOH 1 mM (100 μL) was used as a positive control for ROS formation in 1 of the wells, while ascorbic acid (100 μL of 3 μg/mL solution) was used as an antioxidant control. The plate was incubated for 45 min as previously. Fluorescence was measured at 485 nm/528 nm (BioTek Synergy HT Multi-Mode Microplate Reader). Experiments were carried out 3 times in triplicate.

To determine cell functionality, the content of uric acid was estimated in HK-2 cells supernatant after treatments with IS + G and beverages using a commercial kit (Spinreact, Girona, Spain). In a microplate, 5 µL of the cell supernatant was mixed with 200 μL of the commercial reagent. The mixture was incubated at 15–25 °C for 10 min. Finally, absorbance was measured at 520 nm using an Epoch 2 Microplate spectrophotometer (BioTek, Winooski, VT, USA). A uric acid standard solution (357 µmol/L) was used for quantification and reaction control. Analyses were carried out 3 times in triplicate, and the results were expressed in µmol/L.

### 2.9. Lipopolysaccharide-Induced Inflammation in RAW 264.7 Macrophages

The anti-inflammatory properties of black tea and coffee cascara infusions and kombuchas were determined by quantifying the nitric oxide (NO) production in macrophages (RAW 264.7), as described by Benayad et al. [61].

RAW 264.7 macrophage cells from murine (ATCC, accession number TIB-71) were part of the BAT Unit collection of Instituto de Investigación en Ciencias de la Alimentación (CIAL, CSIC-UAM, Madrid, Spain). Cells were cultured in DMEM with 4.5 g of glucose and supplemented with 10% FBS, 1% L-glutamine, and 1% penicillin/streptomycin. The cells were incubated in standard conditions (37 °C, 5% CO_2_, in a humidified incubator (BINDER CB series 2010, Tuttlingen, Germany). They were then seeded on a 96-well plate (8 × 10^4^ cell/well) and cultured in complete medium (DMEM with 4.5 g/L of glucose, 10% *v*/*v* of Fetal Bovine Serum (FBS), 1% *v*/*v* of L-glutamine and 1% *v*/*v* of antibiotics) for 24 h (37 °C, 5% CO_2_). Following, cells were treated with 150 µL of FBS-free medium containing 1 µg/mL lipopolysaccharide (LPS) from *Escherichia coli* O55:B5 and the test beverages (100 µL). Then, cells were incubated for 24 h (37 °C, 5% CO_2_). Negative and positive controls consisted of an FBS-free medium and 1 µg/mL of LPS in an FBS-free medium, respectively. After the incubation period, 100 µL of supernatants from the wells were removed and combined with 100 µL of Griess reagent (1% *w*/*v* sulfanilamide and 0.1% *w*/*v* N-1-(naphthyl)-ethylenediaminedihydrochloride in 2.5% *v*/*v* H_3_PO_4_). The mixtures were incubated at room temperature in the dark for 15 min, and absorbance was measured at 550 nm in a BioTek Epoch 2 Microplate spectrophotometer (Winooski, VT, USA). A calibration curve with NO in FBS-free DMEM was used for quantification (0–10 µg/mL). Experiments were carried out 3 times in triplicate.

### 2.10. Statistics

Data are reported as means ± standard deviations. An analysis of variance (ANOVA), followed by Tukey’s test, was performed using GraphPad Prism (Version 8.4.2, Informer Technologies, Los Angeles, CA, USA) to determine significant differences between samples at *p* ≤ 0.05.

## 3. Results and Discussion

### 3.1. Microbial Taxonomy

**Figure 2:**
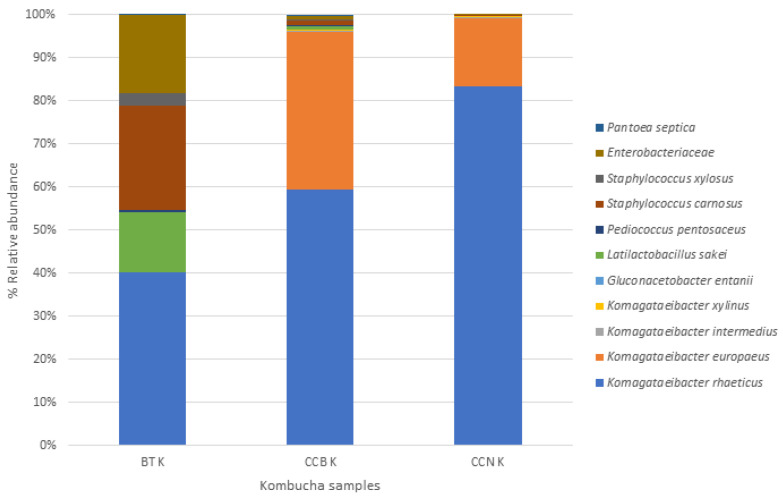
Bacterial composition of the solid and liquid phases of the black tea kombucha starter and coffee cascara kombuchas (9 days of fermentation) consortia. Note: BT K: black tea kombucha; CCB K: kombucha made with coffee cascara from Brazil; CCN K: Kombucha made with coffee cascara from Nicaragua.

This analysis was performed to characterize the consortium and allow reproducibility, considering that the SCOBY composition may differ worldwide and that microorganisms may contribute differently to the changes in the chemical composition and physiological effects of kombucha, although all SCOBYs contain mainly acetic acid bacteria and yeasts, in addition to minor components [46,62]. The microbial community of the starter culture and the final liquid and biofilms from coffee cascara kombuchas (at day 9) were evaluated (Figure 2). Data analysis of the 16S rRNA gene sequence revealed two bacterial phyla in all samples, Proteobacteria and Firmicutes. Proteobacteria (members of this phylum are *Komagataeibacter* and *Gluconacetobacter*) was the most abundant phyla, especially in coffee cascara kombuchas, with a percentage higher than 90%. These results agree with previous studies that analyzed the microbial composition of kombucha beverages [18,63,64].

In all kombucha beverages, the most abundant genus observed in the liquid and biofilm was *Komagataeibacter*, a genus of acetic acid bacteria and the most efficient bacterial cellulose producer. This is also in accordance with previous studies characterizing kombucha cultures [18]. In the starter culture (black tea kombucha), only the specie *Komagataeibacter rhaeticus* was identified, representing about 40% of the total bacteria. These are known to be some of the most abundant bacterial members among the kombucha fermenting agents [46,65]. Still, in black tea kombucha, high percentages of *Staphylococcus* (24%), Enterobacteriaceae (18%), *Latilactobacillus sakei* (15%), and a low percentage of *Pediococcus pentosaceus* (0.4%) were observed.

*K. xylinus* was identified only in both types of coffee cascara kombuchas (0.02%). This species was previously identified in *C. sinensis* kombuchas [65]. Lavasani et al. [66] isolated *K. xylinus* from apple vinegar, and this strain survived in acid and bile environments, demonstrating a potential probiotic effect. Two lactic acid bacteria were identified in black tea kombucha and CCB kombucha, *Latilactobacillus sakei* and *P. pentosaceus*. The Lactobacillae genus was previously identified in kombucha liquid and pellicle [64,65]. These strains are potentially probiotic bacteria [67,68].

In the present study, members of the Enterobacteriaceae family were identified in liquid and pellicle samples, with a higher percentage in black tea kombucha (18%) and a very low percentage in coffee cascara kombuchas (<0.6%). This family has been previously identified in *C. sinensis* kombucha liquid or pellicle [46]. They are important; while some of these organisms are involved in food spoilage, some are indicators of fecal contamination of food products, and others are food-borne pathogens [69]. These bacteria were not viable since pre-boiling water was used to prepare the infusion to make kombucha [26].

**Figure 3:**
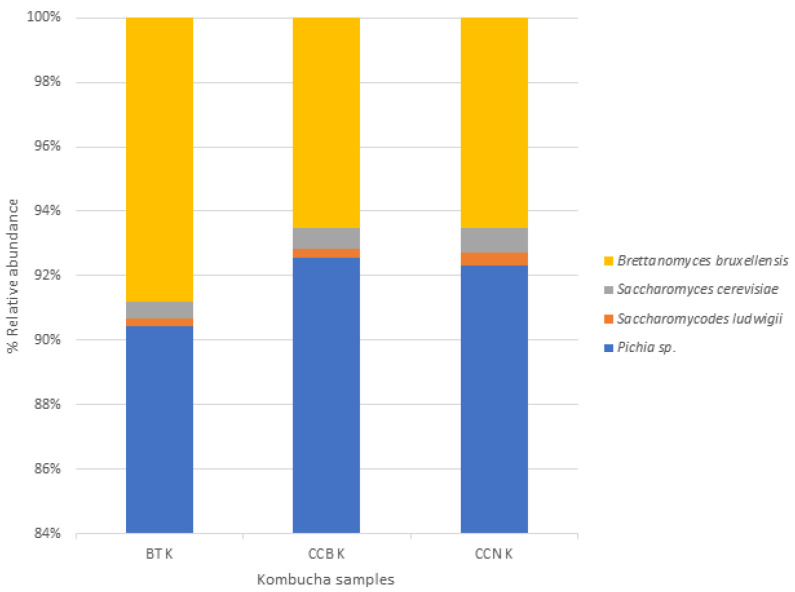
Yeast composition of the solid and liquid phases of black tea kombucha starter and coffee cascara kombuchas after 9 days of fermentation. Note: BT K: black tea kombucha; CCB K: kombucha made with coffee cascara from Brazil; CCN K: kombucha made with coffee cascara from Nicaragua.

Regarding yeasts, data analysis of ITS1 identified that the most abundant phyla was Ascomycota (Figure 3). *Pichia* (the predominant yeast genera with an abundance higher than 70%), followed by *Saccharomyces* (>2%). *Brettanomyces bruxellensis* (5%) strain was present in all kombuchas. Another non-saccharomyces strain comprised 0.4% of the total yeasts. *Saccharomyces cerevisiae* is invariably the dominant species in the winemaking and fermentation process [70]. Non-saccharomyces yeasts from *Pichia* genera are also potential probiotics strains [71], and *Saccharomycodes ludwigi* can be a spoilage yeast in wine, conferring to it undesirable flavors [72]. *Brettanomyces bruxellensis* is the most common yeast identified in kombucha tea and SCOBY [65,73]. It has also been identified in the starter and *C. sinensis* kombuchas. In kombuchas, *B. bruxellensis* can stimulate acetic acid bacteria biofilm production [65].

Many kombucha benefits are attributed to the interaction of microorganisms with phenolic compounds in tea composition. The metabolism of microorganisms during kombucha fermentation is complex. It has yet to be fully elucidated due to the large number of microorganisms involved and the interactions that occur among them [8]. Infusions fermentation by the kombucha consortium tends to increase their bioactive potential and promote the synergy between the fermentation metabolites and the microorganisms [15].

### 3.2. Beverages Characterization

#### 3.2.1. pH, Total Acidity, Soluble Solids, and Sugars

**Figure 4:**
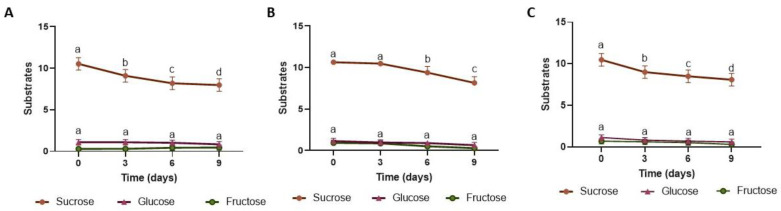
Content of sucrose, glucose, and fructose (g/100 mL) in BT K (**A**), CCB K (**B**) and CCN K (**C**) from day 0 to day 9 of fermentation. Data are presented as the means of triplicate analysis ± standard deviation. BT: Black tea; CCB: Coffee cascara from Brazil; CCN: Coffee cascara from Nicaragua. K: kombucha. Different letters on the same line indicate statistical differences by ANOVA followed by Tukey’s test (*p* < 0.05).

The pH, total acidity, and content of soluble solids in all infusions and fermented beverages are presented in Table 1. An increase in soluble solids values occurred from infusions to day 0 because of the addition of other kombucha components. Acidity also increased, and pH decreased on day 0 because of the addition of the starter culture to promote the adequate pH for the symbiotic culture to develop and ferment. As fermentation proceeded from day 0 to day 9, soluble solids values decreased (11.6–9.3 °Brix, Table 1). Such a decrease is mostly associated with changes in sugar concentration in the culture medium overtime (Figure 4) [74]. During fermentation, part of the sucrose was degraded, with an initial concentration of 10 g/100 mL on day 0 and 8–8.2 g/100 mL on day 9 (Figure 4). Such a decrease in sugar content was not remarkable, probably because of the sugar content in the cascara infusion (about 1% by weight). In this process, yeast and bacteria produce invertase which cleaves the disaccharide sucrose to its monosaccharide components, glucose and fructose [75]. The activity of this enzyme increases along fermentation [76]. No significant difference was found in glucose (0.62–1.14 g/100 mL) and fructose (0.25–0.93 g/100 mL) concentrations in the fermented medium because both monosaccharides were consumed, with no accumulation, given the multiple microorganisms and biochemical pathways co-occurring [15], including the formation of organic acids, mainly acetic acid [77]. The values of soluble solids, titratable acidity, and pH are within the range previously observed in kombucha beverages in general [17,78] and higher than the pH found by Muzaifa et al. [44] for a fermented cascara infusion (2.6–3.1).

It is worth noting that the amount of sugar used in this study was classically used worldwide, and it was chosen because Brazilians still like sweet foods, although there is a joint effort to try to change this habit due to the new world trends aligned with scientific advancements in public health. The current average sugar content in kombuchas found in the American and European markets is within the range of 2–6% (by weight). This is a great alternative to traditional soft drinks, which usually contain more than 10% sugar (by weight) and no nutrients or bioactive compounds ([21], unpublished market research). This alternative could be especially beneficial to young adults and teenagers, who are the greatest soft drink/soda consumers worldwide [79,80,81].

#### 3.2.2. Bioactive Compounds

##### Black Tea

**Figure 5:**
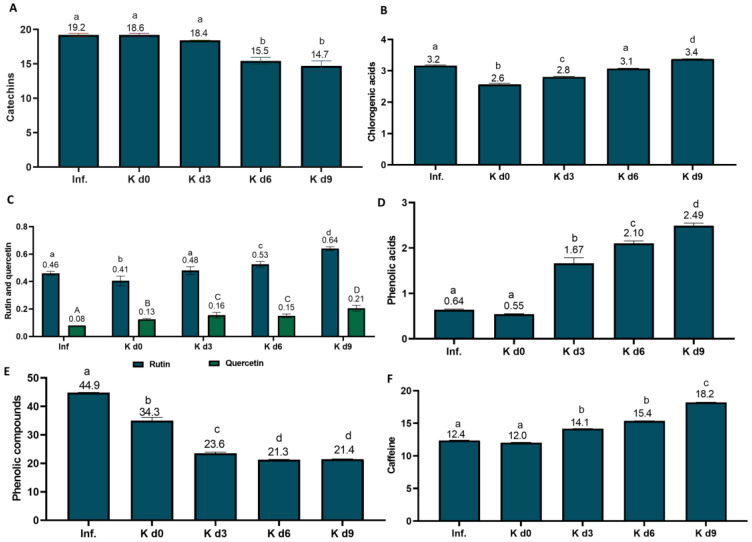
Content of bioactive compounds (mg/100 mL) in black tea infusion and kombuchas. Results are the means of duplicate analysis. Different letters over the bars for each bioactive compound indicate statistical difference (*p* < 0.05) by ANOVA, followed by Tukey’s test. Inf: infusion; K d0: kombucha on day 0; K d3: kombucha on day 3; K d6: kombucha on day 6; K d9: kombucha on day 9. (**A**) Total catechins—sum of the catechins epigallocatechin gallate; gallocatechin; epigallocatechin gallate; gallocatechin gallate; and epicatechin; (**B**) Total CGA—sum of3-caffeoylquinic acid; 4-caffeoylquinic acid; 5-caffeoylquinic acid; (**C**) rutin and quercetin; (**D**) sum of all phenolic acids—caffeic acid, gallic acid; 3,4-dihydroxybenzoic acid; hippuric acid; 3,4-dihydroxyphenylacetic acid; 4-hydroxyphenylacetic acid, vanillic acid; dihydrocaffeic acid; (**E**) sum of all quantified phenolic compounds; (**F**) caffeine.

Three major catechins were identified in the black tea infusions and kombuchas: epigallocatechin gallate, epicatechin, and epigallocatechin, with the latter being the most abundant. Four corresponding minor stereoisomers were identified, including gallocatechin gallate, gallocatechin, catechin gallate, and catechin. The concentrations of total catechins in the beverages are presented in Figure 5A. Catechin gallate and gallocatechin gallate were not detected in black tea samples. The content of total catechins in black tea infusion is within the range found by Koch et al. [82] (2.69–35.1 mg/100 mL), for different black teas, according to brewing time. Along fermentation, the content of catechins decreased by 1%, 17%, and 21% after 3, 6, and 9 days, respectively, compared to day 0. Gaggia et al. [83] also observed decreased catechins content during black tea kombucha fermentation.

The total chlorogenic acid concentrations in black tea infusion and kombuchas are presented in Figure 5B. An 18% decrease was observed in total chlorogenic acids from infusion to day 0 because of the dilution with other kombucha components. Then, a 30% increase up to day 9 occurred. Villarreal-Soto et al. [15,46] identified the main chlorogenic acid, 5-caffeoylquinic acid (1.97–1.97 mg/100 mL), in black tea infusion and kombuchas. In the present study, additional chlorogenic acids were identified from day 0 to 9. They were 3-caffeoylquinic acid (0.51–0.86 mg/100 mL) and 4-caffeoylquinic acid (0.60–0.55 mg/100 mL). The compounds 3-feruloylquinic acid, 4-feruloylquinic acid, 5-feruloylquinic acid, 3,4-dicaffeoylquinic acid, 3,5-dicaffeoylquinic acid and 4,5-dicaffeoylquinic acid were not identified in black tea beverages.

Rutin and quercetin were additional flavonoids identified in black tea beverages, as reported in previous studies [15,46]. Their concentration increased as fermentation progressed (a 56–61% increase from day 0 to day 9, respectively; Figure 5C). This increase is mainly attributed to the breakage between aglycones and glucosides by esterases [84] produced by the symbiotic consortium and by the degradation of other flavonoids, including catechins, in the case of rutin [15,46].

The content of free phenolic acids increased by 353% in black tea kombucha beverages from day 0 to 9 (Figure 5D). Gallic acid, a microbial metabolite of catechins and chlorogenic acids [85,86], was identified in black tea infusion. Then, the concentration increased with fermentation (0.55–0.88 mg/100 mL). Gallic acid has previously been identified in black tea infusion and kombuchas [15,46]. It derives from the degradation of catechins and other phenolic compounds like chlorogenic acids. The concentration of caffeic acid (identified from day 3) increased on day 6 (0.08–0.12 mg/100 mL) and decreased on day 9 (0.10 mg/100 mL). Other phenolic acids were identified on day 3 and increased up to day 9; for example, dihydrocaffeic acid (0.12–0.46 mg/100 mL), vanillic acid (0.21–0.30 mg/100 mL), hippuric acid (0.31–0.44 mg/100 mL), 4-hydroxyphenylacetic acid (0.11–0.15 mg/100 mL), and 3,4-dihydroxyphenylacetic acid (0.15–0.17 mg/100 mL).These compounds are known as colonic metabolites of chlorogenic acids and other phenolic compounds [86]. The main colonic metabolites from catechins, epigallocatechin and epicatechin are supposedly (-)-5-(3′,4′,5′-trihydroxyphenyl)-γ-valerolactone and (-)-5-(3′,4′-dihydroxyphenyl)-γ-valerolactone [87], but they were not quantified in this study due to the lack of standards. Despite the increase in free aglycones and metabolites, there was a 38% decrease in the major phenolic compound’s concentration by the end of fermentation (Figure 5E), as also observed by Villarreal-Soto et al. [15]. However, some oxidized compounds, like the theaflavins contained in black tea [88], were not quantified.

In the present study, the concentration of caffeine (Figure 5F) increased during kombucha fermentation (51% from day 0 to day 9), probably due to the release from complexes with phenolic compounds and other biomolecules, for example, 5-caffeoylquinic acid [89], during fermentation. To our knowledge, this is the first time that an increase in total chlorogenic acids has been reported during kombucha fermentation [15,46].

##### Coffee Cascara

**Figure 6:**
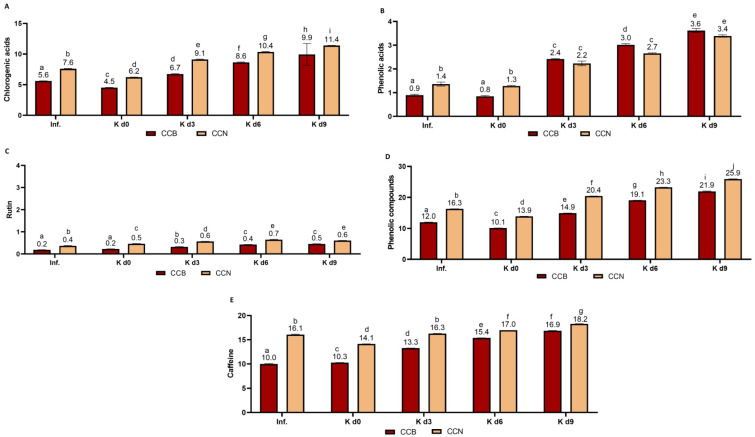
Concentrations (mg/100 mL) of bioactive compounds in coffee cascara infusions and kombuchas (CCB: Coffee cascara from Brazil; CCN: Coffee cascara from Nicaragua). Results are the means of duplicate analysis. Different letters between samples for each bioactive compound indicate significant differences (*p* ≤ 0.05) by ANOVA followed by Tukey’s test. Inf: infusion; K d0: kombucha on day 0; K d3: kombucha on day 3; K d6: kombucha on day 6; K d9: kombucha on day 9 (**A**) total chlorogenic acids: 3-caffeoylquinic acid; 4-caffeoylquinic acid; 5-caffeoylquinic acid; 4-feruloylquinic acid; 5-feruloylquinic acid; 3,4-dicaffeoylquinic acid; 3,5-dicaffeoylquinic acid; 4,5-dicaffeoylquinic acid; (**B**) total phenolic acids: caffeic acid, gallic acid; ferulic acid; p-coumaric acid; benzoic acid; 3,4-dihidroxybenzoic acid; hippuric acid; 3,4-dihidroxyphenylacetic acid; 4-hidroxyphenylacetic acid, vanillic acid; dihydrocaffeic acid; (**C**) rutin and quercetin; (**D**) sum of all quantified phenolic compounds; (**E**) caffeine.

Eight chlorogenic acids were identified in coffee cascara infusions and kombuchas (Figure 6A). Higher chlorogenic acids content in CCN infusion (7.6 mg/100 mL) was observed compared to CCB (5.6 mg/100 mL) for different reasons, including plant variety, edapho-climatic conditions, and agricultural practices, such as post-harvest processing methods. While CCN went through wet-processing, which includes a fermentative phase that can release bound chlorogenic acids [90], CCB was dry-processed.

The total chlorogenic acids concentration decreased by 19% (the average of CCB and CCN) from infusions to day 0 because of dilution caused by the addition of other ingredients. In general, there was a considerable increase in total concentrations along fermentation, especially in 4-feruloylquinic acid (0.08–0.53 mg/100 mL), 4-caffeoylquinic acid (0.25–1.16 mg/100 mL), and 3-caffeoylquinic acid (0.34–1.27 mg/100 mL) from day 0 to 9. The concentration of 5-caffeoylquinic acid increased in both cascara kombuchas (3.63–6.06 mg/100 mL, on average) from day 0 to 9. Slight decreases in 3,4-dicaffeoylquinic acid (0.12–0.10 mg/100 mL), 4,5-dicaffeoylquinic acid (0.19–0.17 mg/100 mL), and 3,5-dicaffeoylquinic acid (0.58–0.54 mg/100 mL) were observed. The total mean concentration of chlorogenic acids increased 98% along fermentation, up to day 9, with a higher increase in CCB. This increase is probably derived from the action of enzymes from the symbiotic consortium on chlorogenic acids molecules, given that some species of microorganisms contain esterases capable of hydrolyzing chlorogenic acid to caffeic and quinic acids [91,92].

The main phenolic acids identified in coffee cascara kombuchas were gallic acid (0.29–0.52 mg/100 mL), caffeic acid (0.14–0.29 mg/100 mL), ferulic acid (0.13–0.29 mg/100 mL), 3,4-dihydroxybenzoic acid (0.21–0.37 mg/100 mL), benzoic acid (0.11 –0.16 mg/100 mL), p-coumaric acid (0.11–0.16 mg/100 mL), hippuric acid (0.20–0.39 mg/100 mL), 3,4-dihydroxyphenylacetic acid (0.16–0.28 mg/100 mL), vanillic acid (0.28–0.42 mg/100 mL), and dihydrocaffeic acid (0.21–0.40 mg/100 mL), with the latest five compounds identified only from day 3 to 9. These compounds are known as colonic metabolites of chlorogenic acids [89]. They were identified in urine and plasma samples after coffee consumption [56]. The content of phenolic acids (Figure 6B) increased by 218%, on average, from day 0 to 9, due to the degradation of chlorogenic acids and other phenolic compounds.

Rutin (Figure 6C) was also identified in coffee cascara beverages, with a higher concentration in kombuchas (50% average increase from day 0 to 9, on average), although the content in cascara beverages was lower than in black tea infusion and kombuchas. Heeger et al. [36] have also identified rutin in cascara teas. Although rutin can be metabolized to quercetin by microorganisms [93], only traces of quercetin (<0.001–<0.003 mg/100 mL) were identified in fermented coffee cascara beverages.

In coffee cascara beverages, trace concentrations of seven catechins (epigallocatechin, gallocatechin, epigallocatechin gallate, gallocatechin gallate, epicatechin, catechin) were identified (<0.02 mg/100 g-LOQ). Ramirez–Martinez et al. [94] and Mullen et al. [95] found 2–26 mg/100 g of catechin and 1–264 mg/100 g of epicatechin in the fresh coffee pulp. On the other hand, Heeger et al. [36] did not detect catechin or epicatechin in coffee cascara and pulp. According to the authors, epicatechin may be degraded during processing and storage, which could have been the case in this study. The total free phenolic compounds content in coffee cascara kombuchas increased 116% in CCB and 86% in CCN from day 0 to 9 (Figure 6D). These are higher percentages compared to black tea.

Caffeine was identified in all coffee cascara beverages (10–18 mg/100 mL; Figure 6E), increasing, on average, 44% from day 0 to 9. Heeger et al. [36] found 22.6 mg/100 mL in a cascara infusion. As aforementioned, the caffeine-chlorogenic acid complex, mainly with 5-caffeoylquinic acid [89], was probably hydrolyzed by kombucha consortia and can explain the caffeine increase, in addition to chlorogenic acid’s increase.

### 3.3. Potential Antioxidant Effect of Black Tea and Coffee Cascara Beverages in Indoxyl Sulfate-Induced HK-2 Cells

HK-2 cells were treated with IS and high G to increase oxidative stress (ROS production) and simulate the condition of diabetic nephropathy. IS is known as a gut-derived uremic toxin, which induces free radical production in renal tubular and glomerular mesangial cells [96,97]. In normal kidney proximal tubular epithelial cells, IS has been shown to reduce proliferation leading to cellular senescence. It adversely affects redox control and mitochondrial metabolism and promotes pro-fibrotic/inflammatory gene expression [98], leading the cells to apoptosis, hypertrophy, mitochondrial dysfunction, and pro-fibrotic and inflammatory molecules [99]. Coffee cascara extract has previously reduced (*p* < 0.05) the expression of TNFα, NLRP 3, and CCL 2 when induced by IS associated with G or fructose 25 mM [60].

Cells viability measurement was performed to evaluate the cytotoxicity of the tested solutions and beverages and to normalize the results considering the viable cells. DMSO (50%; positive death control) caused death in 94% of the cells, as expected [100], compared to DMEM (life control). The administration of IS + G decreased cell viability by 30%. The association of IS + G with black tea beverages increased viability by 80%, while the association with coffee cascara did not increase viability significantly. The present results showed that the cells treated with the test beverages were viable during the experiment, with higher viability for black tea cells. The detailed results are presented as Supplementary material (Figure S1).

**Figure 7:**
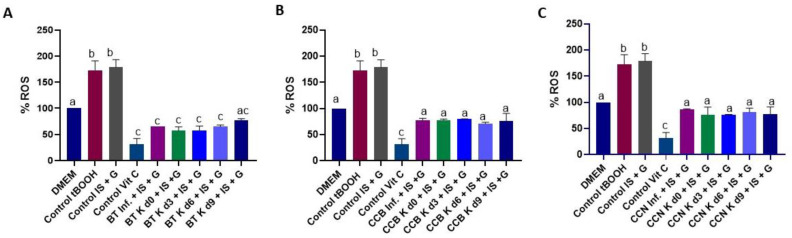
Effect of BT (**A**), CCB (**B**) or CCN (**C**) beverages, associated with IS and G, on ROS formation, determined by fluorescence, in nephropaticHK2 cells. tBOOH: tert-Butyl hydroperoxide (oxidant control); Vit C: ascorbic acid (antioxidant control); IS: indoxyl sulfate; G: culture media with high glucose (25 mM) solution; d0, d3, d6, and d9: days 0, 3, 6, and 9 of fermentation, respectively. BT: black tea; Inf: plain infusion; K: kombucha; CCB: Coffee cascara from Brazil; CCN: Coffee cascara from Nicaragua. Different letters over the bars indicate statistical differences among treatments by ANOVA followed by Tukey’s test (*p* < 0.05).

The induced (nephropathic) cells were submitted to the test infusions and kombuchas. The results are presented in Figure 7. tBOOH 1 mM, an oxidative agent used as a positive control, induced ROS production compared to DMEM (negative control). As expected, IS + G treatment also induced ROS production. Treating HK-2 cells with black tea infusion and kombuchas associated with IS + high G suppressed (*p* < 0.0001) ROS production in 32–42%. Coffee cascara tea infusions and kombuchas were also able to reverse (*p* < 0.0001) the effect of oxidative damage caused by the combined addition of IS and high G, with a 43% reduction in ROS production, on average, with no statistical difference when compared to black tea beverages. Although sugar could potentially antagonize the antioxidant effect of the beverage due to its ability to induce ROS generation [101], the results were similar for the infusions and kombuchas, which means that possibly the kombucha compounds have also reversed the effect of sugar. No difference was observed in the results during the period of fermentation.

Black tea kombucha decreased ROS generation in a mouse hepatocyte after the induction of oxidative stress with tertiary butyl hydroperoxide [12]. Bhattacharya et al. [102] have also demonstrated the protective effect of black tea kombucha against oxidative stress-mediated damage in different tissues of diabetic rats. The suppression of ROS production in HK-2 cells by green *C. sinensis* tea has also been previously reported by Sun et al. [103]. To date, this appears to be the first study evaluating the effect of black tea and cascara tea infusions and kombuchas in the HK-2 cell model of oxidative stress.

We can attribute the reduction in intracellular ROS formation mainly to the polyphenols in black tea and coffee cascara tea. These compounds and their primary metabolites exert antioxidant activity suppressing ROS formation by either inhibiting enzymes involved in their production, scavenging a wide range of ROS in vitro and in vivo, upregulating antioxidant enzymes such as superoxide dismutase, catalase, and glutathione peroxidase [104,105] or reducing the catalytic activity of enzymes involved in ROS generation [86,106,107]. Catechins are constituents of green and black *C. sinensis* tea. These compounds can inhibit prooxidant enzymes, e.g., NADPH (nicotinamide adenine dinucleotide phosphate)-oxidase, or modulate interactions of ligands with receptors, e.g., tumor necrosis factor-alpha (TNF-α). They can also suppress many oxidative stress-related pathways responsible for the inflammation processes. For example, catechins modulate the activities of redox-sensitive transcription factors such as nuclear factor kappabeta (NF-κB) and activator protein-1 (AP-1), which are very important in response to pathogenesis-related oxidative stress [107]. Several cell culture experiments have also shown the antioxidative properties of chlorogenic acids at both cellular and molecular levels [86]. As a source of chlorogenic acid, coffee cascara has previously prevented intracellular ROS formation in HepG2 cells [27], in RAW 264.7 cells [108,109], and in 3T3-L1 adipocytes [109].

Caffeine might contribute to the overall antioxidant capacity of coffee, as well as its metabolites, especially 1-methylxanthine and 1-methylurate. Previous studies have demonstrated the protective effect of caffeine and/or its metabolites in cell membranes against oxidative damage and LDL peroxidation, in addition to plasma iron-reducing capacity in human subjects after regular coffee consumption [110,111]. Uric acid (UA) production and metabolism are complex processes involving various factors that regulate hepatic production and renal and gut excretion of this compound. UA is the end product of an exogenous pool of purines and endogenous purine metabolism. The exogenous pool varies significantly with diet; animal proteins contribute significantly to this purine pool. The endogenous production of uric acid is mainly from the liver, intestines, and other tissues like muscles, kidneys, and the vascular endothelium [112]. In the present experiment, we used UA as a marker for cell damage. UA is also a urine biomarker for oxidative stress and plays an important role in diabetic nephropathy development. In patients diagnosed with type 2 diabetes, increased UA levels may be an important predictor of nephropathy in diabetic patients [113,114]. In the same way, reducing UA levels in diabetic patients can also reduce the progression of chronic kidney disease [114].

**Figure 8:**
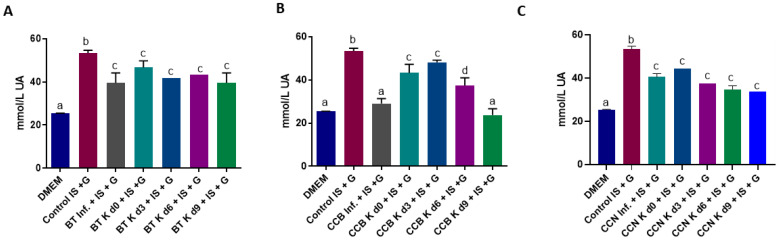
Effect of BT (**A**), CCB (**B**) or CCN (**C**) beverages, associated with IS and G, on uric acid concentration in nephropathicHK-2 cells. Results are the averages of triplicate experiments performed three times. Different letters over the bars indicate significant differences among treatments by ANOVA, followed by Tukey’s test (*p* < 0.05).DMEM: Dulbecco’s Modified Eagle Media; IS: indoxyl sulfate; G: culture media with high glucose solution; d0, d3, d6 and d9: days 0, 3, 6 and 9 of fermentation, respectively; BT: black tea; Inf: plain infusion; K: kombucha; CCB: coffee cascara from Brazil; CCN: coffee cascara from Nicaragua.

When cells were treated with IS, an increase in the concentration of supernatant UA was observed, as expected (Figure 8), given that IS and other uremic toxins concentrations correlate inversely with renal function [115]. DMEM treatment did not affect UA concentrations in the supernatant of the cells. Black tea and cascara infusions reduced 24–45% UA in cell supernatant, with a higher decrease in CCB infusion. Black tea kombuchas reduced in 13–26% the concentration of UA in the supernatants, from day 0 to 9, compared to the cells treated with IS, while cascara beverages reduced the UA concentration by 10–55%. UA concentrations in the supernatant are higher than those found by Hou et al. [116], who used cultured HK-2 cells to establish a stable model of hyperuricemia for long-term studies.

In an in vitro test using xanthine oxidase to increase UA and ROS production, Jayabalan et al. [11] observed that green and black tea infusions and kombuchas could scavenge superoxide radicals, showing significant antioxidant activity. The combined effect of catechins and other polyphenols in black tea infusion and kombuchas can explain the reduction in UA levels compared with cells treated with IS + G.

Polyphenols such as epigallocatechin gallate, quercetin, gallic acid, caffeic acid, chlorogenic acids, and sinapic acid, among others, exert benefit in hyperuricemia, inhibiting the UA enzymes producers (xanthine oxidase and adenosine deaminase) and increasing the excretion of UA [117].Using a murine hyperuricemic model, Zhou et al. [118] observed a decrease in serum UA concentration after administration of 30 or 60 mg/kg of 5-caffeoylquinic acid, the main chlorogenic acid compound in coffee cascara, suggesting that supplementation with this compound could effectively prevent hyperuricemia and mitigate kidney impairment. The high content of chlorogenic acids in coffee cascara kombuchas can explain the decrease in uric acid concentration in cell supernatant. Additionally, caffeine intake through food has been negatively associated with uric acid concentration [119].

### 3.4. Effect of Black Tea and Coffee Cascara Beverages on Anti-Inflammatory Activity in Lipopolysaccharide-Induced RAW 264.7 Cell Line

Regarding cell viability, treating the cells with DMSO (50%; death control) caused death in 95% of cells, compared to DMEM, as expected [100]. In all treatments, plain LPS did not affect the viability of cells significantly. Likewise, the association of black tea with LPS did not affect viability. A slight (9%) decrease in viability of cells treated with coffee cascara infusions and LPS was observed compared to plain LPS, which is negligible. The results are presented as supplementary material (Figure S2).

**Figure 9:**
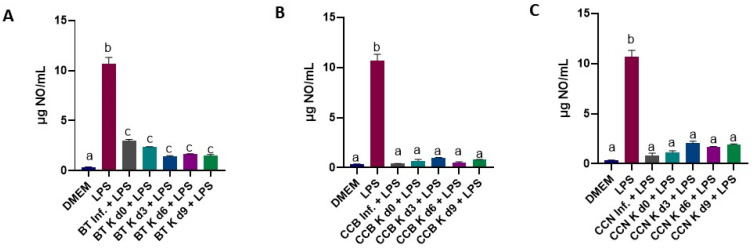
Effect of treatment with BT (**A**), CCB (**B**) or CCN (**C**) beverages, associated with LPS on NO production in RAW 264.7 cells, determined by Griess Reagent. Results are the means of triplicates of experiments performed three times. DMEM: Dubecco’s Modified Eagle Media; LPS: lipopolysaccharide (nitric oxide production control); d0, d3, d6, and d9: days 0, 3, 6, and 9 of fermentation, respectively; BT: black tea; Inf: plain infusion; K: kombucha; CCB: coffee cascara from Brazil; CCN: coffee cascara from Nicaragua. Different letters over the bars indicate statistical differences among treatments by ANOVA followed by Tukey’s test (*p* ≤ 0.05).

The effect of the test beverages on NO production in RAW 264.7 cells induced by LPS is shown in Figure 9. Inflammation is an overactive immune response to harmful cell stimuli. In the inflammatory response, activated macrophages produce large amounts of inflammatory factors such as interleukin-6 (IL-6), tumor necrosis factor-alpha (TNF-α), and nitric oxide (NO) to induce tissue injury at the inflammatory site [120]. Although macrophages are essential for the effective control and clearance of infections, removal of derbies and dead cells, promotions of tissue repair, and wound healing, they also contribute to tissue damage and pathology during infections and inflammatory diseases [121], resulting in a causal association of macrophages with disease states, such as fibrosis, obesity, and cancer [122].Also, macrophages are key inflammatory cells mediating kidney inflammation in experimental and human diabetes [123].

NO, the smallest cell signaling molecule, participates in diverse physiological functions, such as vasodilation, neural transmission, and immune responses [124]. LPS is a component of the cell wall of Gram-negative bacteria. It is a potent activator of the inflammatory response and has a marked stimulatory effect on the immune system. Consequently, small amounts of LPS in the blood due to bacterial infection are sufficient to elicit an inflammatory response through the interaction with toll-like receptors [125].

In this experiment, LPS (positive control) showed the highest (*p* < 0.0001) NO production among the studied samples, as expected (Figure 9). In cells treated with coffee cascara infusions and kombuchas + LPS, NO production was similar (5% higher, with *p* > 0.05) to DMEM control cells, while in cells treated with black tea infusion and kombuchas + LPS, it was 24% higher than in DMEM cells (*p* < 0.0001). Compared to plain LPS, NO production was 81% and 90% lower for black tea beverages and cascara beverages, respectively, suggesting a potential anti-inflammatory effect. Comparing all treatments, no difference was found between infusions and kombuchas made with the same food material. (Figure 9). Slightly but significantly (*p* < 0.0001) lower NO production was observed in cascara beverages compared to black tea beverages.

NO plays a key role in the development of inflammatory diseases involved in the immune response produced by cytokine-activated macrophages [126]. Several in vitro and animal studies have used NO production associated or not with other inflammation biomarkers to evaluate the potential anti-inflammatory activity of different plant foods, including *C. sinensis* beverages. In a study by Lin et al. [127], different types of *C. sinensis* leaf tea extracts strongly inhibited NO production in LPS-induced RAW 264.7. Villarreal-Soto et al. [15] evaluated the anti-inflammatory potential of black tea infusion and kombucha against the enzyme 5-LOX and suggested that kombucha improved the anti-inflammatory effect of the infusion. Novilla et al. [128] evaluated the effect of oolong *C. sinensis* infusion and kombucha on NO production, also using LPS-induced RAW 264.7, and reported NO reduction, similar to the present study. They also observed the reduction of the production of COX-2 and the pro-inflammatory cytokines IL-6, IL-1β and TNF-α. In the study by Wang et al. [129], a traditional black tea kombucha improved the survival status in LPS-treated mice, effectively inhibiting the release of IL-6, IL-1β, and TNF-α, restoring the levels of T cells and macrophages, and down-regulating the signaling pathway of NF-κB transcription factor in mice with LPS-induced sepsis, exhibiting a potential effect against the development of systemic inflammatory responses associated with sepsis.

Studies investigating the potential anti-inflammatory effect of coffee cascara infusion and extract using LPS-induced RAW 264.7 macrophage have also been performed. In two studies by Rebollo-Hernanz et al. [108,109], coffee cascara aqueous extract reduced NO production and the expression of the inflammatory enzymes iNOS and COX-2. Using the human gastric epithelial cells CRL-1739, Magoni et al. [37] studied the anti-inflammatory activity of hydro-alcoholic and aqueous coffee pulp extracts by measurement of IL-8, one of the most important chemokines involved in gastric inflammation release in human gastric epithelial cells. All coffee pulp extracts similarly inhibited IL-8 release. As aforementioned, no study using fermented or unfermented coffee cascara tea was found.

The results obtained in the present study confirm the previous reports on the anti-inflammatory properties of black tea and coffee cascara tea infusions and black tea kombucha and indicate that despite the chemical changes in those food matrices during fermentation, the effect of NO production suppression was preserved, as it did for the ROS production.

The study by Vázquez–Cabral et al. [130] and several other studies using LPS-induced RAW 264.7 cells attributed the anti-inflammatory effect of kombuchas to their polyphenols content. Quercetin and quercetrin, for example, were able to decrease the production of NO and downregulate TNF-α, IL-1β, and IL-6 [131]. 3-OH Flavone, kaempferol, and quercetin were effective in preventing NO production, even when LPS was combined with a heme oxygenase inducer, and did not increase the expression of COX-2 [132]. Novilla et al. [128] reported similar effects of epigallocatechin gallate on NO production, COX-2, IL-6, IL-1b, and TNF-α. Several other studies reported that polyphenols from pulp extracts, by-products, and beers derived from cranberry, black raspberry, red raspberry, strawberry, blueberry, blackberry, cocoa, and citrus inhibited NO production [109,133,134,135,136]. Caffeine has also shown anti-inflammatory effects in LPS-induced RAW 264.7 cells, in a dose-dependent manner, suppressing the level of NO production, inhibiting the release of COX-2 and IL-6, IL-3 and IL-12, and suppressing NF-kβ activation and p38MAPK phosphorylation [137].

## 4. Conclusions

In summary, coffee cascara and black tea infusions and kombuchas suppressed the oxidative stress in HK-2 cells treated with IS and high G concentration and reduced UA concentration in the cell supernatant, similarly. Comparable anti-inflammatory effect was also observed in LPS-treated RAW 264.7 cells after incubation with all tested beverages. The phenolic compounds, including catechins, rutin, quercetin and chlorogenic acids, with the possible support of caffeine, were most likely to be the main compounds responsible for the beneficial effects of these beverages.

It is worth noting that independently of being free, bound, or partly metabolized, all phenolic compounds seem to have exerted a joint effect in the cell models, with no distinction of molecular structures. The health outcomes of this fact should be investigated in future studies, as well as the effect of the different test beverages against clinically relevant proinflammatory cytokines.

Overall, the present results suggest that coffee cascara is a novel promising ingredient for kombucha elaboration with potential health benefits. In addition to supporting sustainable coffee production, coffee cascara kombucha is an alternative functional beverage for consumers to choose over traditional soft drinks, especially for young adults and teenagers, who are the main consumers of this type of beverage. It contains bioactive compounds and nutrients along with a basic hydration purpose and can also be produced with a lower amount of sugar than traditional soft drinks.

## Figures and Tables

**Table 1:** Physicochemical characteristics of infusions and kombuchas made with black tea and coffee cascara tea.

Samples	Days of Fermentation	Titratable Acidity (mEq/L)	pH	Soluble Solids (°Brix)
Black tea	Infusion	0.02 ± 0.01 ^a^	5.5 ± 0.01 ^a^	1.1 ± 0.12 ^a^
	0	0.1 ± 0.00 ^b^	3.8 ± 0.07 ^b^	10.4 ± 0.07 ^b^
	3	0.2 ± 0.05 ^b^	3.5 ± 0.00 ^b^	10.8 ± 0.14 ^b^
	6	0.3 ± 0.06 ^b^	3.5 ± 0.00 ^b^	9.7 ± 0.00 ^c^
	9	0.3 ± 0.06 ^b^	3.4 ± 0.00 ^b^	9.3 ± 0.28 ^c^
CCB	Infusion	0.04 ± 0.00 ^a^	4.4 ± 0.05 ^a^	1.3 ± 0.12 ^a^
	0	0.1 ± 0.00 ^a^	3.7 ± 0.07 ^b^	11.5 ± 0.63 ^b^
	3	0.2 ± 0.00 ^b^	3.6 ± 0.07 ^b^	11.3 ± 0.00 ^b^
	6	0.2 ± 0.00 ^b^	3.6 ± 0.07 ^b^	10.4 ± 0.14 ^c^
	9	0.2 ± 0.00 ^b^	3.5 ± 0.00 ^b^	10.0 ± 0.42 ^c^
CCN	Infusion	0.04 ± 0.00 ^a^	4.3 ± 0.15 ^a^	1.2 ± 0.15 ^a^
	0	0.04 ± 0.00 ^a^	3.8 ± 0.21 ^b^	11.6 ± 0.14 ^b^
	3	0.2 ± 0.00 ^b^	3.6 ± 0.07 ^b^	10.9 ± 0.14 ^b^
	6	0.2 ± 0.05 ^b^	3.5 ± 0.00 ^b^	10.6 ± 0.49 ^b^
	9	0.4 ± 0.08 ^b^	3.5 ± 0.00 ^b^	9.9 ± 0.56 ^c^

Results are expressed as mean ± standard deviation for three replicate analyses; different letters on the same column for the same beverage indicate a significant difference (*p* < 0.05); CCB: coffee cascara from Brazil; CCN: coffee cascara from Nicaragua.

## Data Availability

The data presented in this study are available on request from the corresponding author.

## Supplementary Materials

The following supporting information can be downloaded at: www.mdpi.com/article/10.3390/foods12091905/s1, Figure S1: Effect of treatment with BT (A), CCB (B) and CCN (C) beverages, associated with IS and high G on HK-2 cells viability, determined by MTT assay. Results are the average of triplicates of experiments performed three times. Different letters over the bars indicate statistical differences among treatments by ANOVA followed by Tukey’s test (*p* < 0.05). DMEM: Dulbecco’s Modified Eagle Media (life control); DMSO: Dimethyl Sulphoxide (death control). IS: indoxyl sulfate; G: culture media with high glucose solution; d0, d3, d6, and d9: days 0, 3, 6, and 9 of fermentation, respectively; BT: black tea; Inf: plain infusion (no starter and sugar); K: kombucha; CCB: coffee cascara from Brazil; CCN: coffee cascara from Nicaragua. Different letters over the bars indicate statistical differences among treatments by ANOVA followed by Tukey’s test (*p* < 0.05); Figure S2: Effect of treatment with BT (A), CCB (B) or CCN (C) beverages associated with LPS, on RAW 264.7 cells viability, determined by MTT assay. Results are the means of triplicates of experiments performed three times. DMEM: Dulbecco’s Modified Eagle Media (life control); DMSO: Dimethyl Sulphoxide (death control). d0, d3, d6 and d9: days 0, 3, 6, and 9 of fermentation, respectively.BT: black tea; Inf: infusion; K: kombucha CCB: Coffee cascara from Brazil; CCN: Coffee cascara from Nicaragua. Different letters over the bars indicate statistical differences among treatments by ANOVA followed by Tukey’s test (*p* < 0.05).

## References

[1] Zhu M.-Z., Lu D.-M., Ouyang J., Zhou F., Huang P.-F., Liu Z.-H. (2020). Tea consumption and colorectal cancer risk: A meta-analysis of prospective cohort studies. Eur. J. Nutr..

[2] Chung M., Zhao N., Wang D., Shams-White M., Karlsen M., Cassidy A., Wallace T.C. (2020). Dose-response relation between tea consumption and risk of cardiovascular disease and all-cause mortality: A systematic review and meta-analysis of population-based studies. Adv. Nutr..

[3] Yang W.-S., Wang W.-Y., Fan W.-Y., Deng Q., Wang X. (2014). Tea consumption and risk of type 2 diabetes: A dose–response meta-analysis of cohort studies. Br. J. Nutr..

[4] Arab L., Khan F., Lam H. (2013). Tea consumption and cardiovascular disease risk. Am. J. Clin. Nutr..

[5] Aboulwafa M.M., Youssef F.S., Gad H.A., Altyar A.E., Al-Azizi M.M., Ashour M.L. (2019). A comprehensive insight on the health benefits and phytoconstituents of *Camellia sinensis* and recent approaches for its quality control. Antioxidants.

[6] Tang G.-Y., Meng X., Gan R.-Y., Zhao C.-N., Liu Q., Feng Y.-B., Li H.-B. (2019). Health functions and related molecular mechanims of tea components: An update review. Int. J. Mol. Sci..

[7] Martínez-Leal J., Suárez L.V., Jayabalan R., Oros J.H., Escalante-Aburto A. (2018). A review on health benefits of kombucha nutritional compounds and metabolites. CyTA-J. Food.

[8] Coelho R.M.D., de Almeida A.L., do Amaral R.Q.G., da Mota R.N., de Sousa P.H.M. (2020). Kombucha: Review. Int. J.Gastron. Food Sci..

[9] Diez-Ozaeta I., Astiazaram O.J. (2022). Recent advances in Kombucha tea: Microbial consortium, chemical parameters, health implications and biocellulose production. Int. J. Food Microbiol..

[10] Greenwalt C.J., Ledford R.A., Steinkraus K.H. (1998). Determination and characterization of the antimicrobial activity of the fermented tea kombucha. LWT—Food Sci. Technol..

[11] Jayabalan R., Subathradevi P., Marimuthu S., Sathishkumar M., Swaminathan K. (2008). Changes in free-radical scavenging ability of kombucha tea during fermentation. Food Chem..

[12] Bhattacharya S., Gachhui R., Sil P.C. (2011). Hepatoprotective properties of kombucha tea against TBHP-induced oxidative stress via suppression of mitochondria dependent apoptosis. Pathophysiology.

[13] Srihari T., Kathikesan K., Ashokkumar N., Satyanarayana U. (2013). Antihyperglycaemic efficacy of kombucha in streptozotocin-induced rats. J. Funct. Foods.

[14] Rahmani R., Beaufort S., Villarreal-Soto S.A., Taillandier P., Bouajila J., Debouba M. (2019). Kombucha fermentation of African mustard (*Brassica tournefortii*) leaves: Chemical composition and bioactivity. Food Biosci..

[15] Villarreal-Soto S.A., Beaufort S., Bouajila J., Souchard J.-P., Renard T., Rollan S., Taillandier P. (2019). Impact of fermentation conditions on the production of bioactive compounds with anticancer, anti-inflammatory and antioxidant properties in kombucha tea extracts. Process. Biochem..

[16] Kaewkod T., Bovonsombut S., Tragoolpua Y. (2019). Efficacy of kombucha obtained from green, oolong, and black teas on tnhibition of pathogenic bacteria, antioxidation and toxicity on colorectal cancer cell line. Microorganisms.

[17] Cardoso R.R., Neto R.O., D’Almeida C.T.S., do Nascimento T.P., Pressete C.G., Azevedo L., Martino H.S.D., Cameron L.C., Ferreira M.S.L., de Barros F.A.R. (2020). Kombuchas from green and black teas have different phenolic profile, which impacts their antioxidant capacities, antibacterial and antiproliferative activities. Food Res. Int..

[18] Chakravorty S., Bhattacharya S., Chatzinotas A., Chakraborty W., Bhattacharya D., Gachhui R. (2016). Kombucha tea fermentation: Microbial and biochemical dynamics. Int. J. Food Microbiol..

[19] Mukherjee A., Gómez-Sala B., O’Connor E., Kenny J.G., Cotter P.D. (2022). Global regulatory frameworks for fermented foods: A review. Front. Nutr..

[20] Grand View Research Kombucha Market Size, Share & Trends Analysis Report by Product (Conventional, Hard), By Distribution Channel (On-Trade, Off-Trade), By Region, and Segment Forecasts, 2022–2030. https://www.grandviewresearch.com/industry-analysis/kombucha-market.

[21] Kim J., Adhikari K. (2020). Current trends in kombucha: Marketing perspectives and the need for improved sensory research. Beverages.

[22] Leonarski E., Guimarães A.C., Cesca K., Poletto P. (2022). Production process and characteristics of kombucha fermented from alternative raw materials. Food Biosci..

[23] Esquivel P., Jiménez V.M. (2012). Functional properties of coffee and coffee by-products. Food Res. Int..

[24] Coffee Production Worldwide from 2003/04 to 2020/21 (In Million 60 Kilogram Bags). https://www.statista.com/statistics/263311/worldwide-production-of-coffee/.

[25] Coffee Production by Exporting Countries. https://www.ico.org/prices/po-production.pdf.

[26] DePaula J., Cunha S., Cruz A., Sales A.L., Revi I., Fernandes J., Ferreira I.M.P.L.V.O., Miguel M.A.L., Farah A. (2022). Volatile fingerprinting and sensory profiles of coffee cascara teas produced in Latin American countries. Foods.

[27] Iriondo-DeHond A., García N.A., Fernandez-Gomez B., Guisantes-Batan E., Escobar F.V., Blanch G.P., San Andres M.I., Sanchez-Fortun S., del Castillo M.D. (2019). Validation of coffee by-products as novel food ingredients. Innov. Food Sci. Emerg. Technol..

[28] Murthy P.S., Naidu M.M. (2012). Recovery of phenolic antioxidants and functional compounds from coffee industry by-products. Food Bioprocess. Technol..

[29] Janissen B., Huynh T. (2018). Chemical composition and value-adding applications of coffee industry byproducts: A review. Resour., Conserv. Recycl..

[30] Prata E.R.B.A., Oliveira L.S. (2007). Fresh coffee husks as potential sources of anthocyanins. LWT.

[31] Rios M.B., Iriondo-DeHond A., Iriondo-DeHond M., Herrera T., Velasco D., Gómez-Alonso S., Callejo M.J., del Castillo M.D. (2020). Effect of coffee cascara dietary fiber on the physicochemical, nutritional and sensory properties of a gluten-free bread formulation. Molecules.

[32] Bondam A.F., da Silveira D.D., dos Santos J.P., Hoffmann J.F. (2022). Phenolic compounds from coffee by-products: Extraction and application in the food and pharmaceutical industries. Trends Food Sci. Technol..

[33] Bobková A., Poláková K., Demianová A., Belej L., Bobko M., Jurcaga L., Gálik B., Novotná I., Iriondo-DeHond A., del Castillo M.D. (2022). Comparative analysis of selected chemical parameters of *Coffea arabica*, from Cascara to Silverskin. Nutrients.

[34] Iriondo-DeHond A., Iriondo-DeHond M., del Castillo M.D. (2020). Applications of compounds from coffee processing by-products. Biomolecules.

[35] Duangjai A., Suphrom N., Wungrath J., Ontawong A., Nuengchamnong N., Yosboonruang A. (2016). Comparison of antioxidant, antimicrobial activities and chemical profiles of three coffee (*Coffea arabica* L.) pulp aqueous extracts. Integr. Med. Res..

[36] Heeger A., Kosinska-Cagnazzo A., Cantergiani E., Andlauer W. (2017). Bioactives of coffee cherry pulp and its utilisation for production of Cascara beverage. Food Chem..

[37] Magoni C., Bruni I., Guzzetti L., Dell’Agli M., Sangiovanni E., Pizza S., Regonesi M.E., Maldini M., Spezzano R., Caruso D. (2018). Valorizing coffee pulp by-products as anti-inflammatory ingredient of food supplements acting on IL-8 release. Food Res. Int..

[38] Khochapong W., Ketnawa S., Ogawa Y., Punbusayakul N. (2021). Effect of in vitro digestion on bioactive compounds, antioxidant and antimicrobial activities of coffee (*Coffea arabica* L.) pulp aqueous extract. Food Chem..

[39] Del Castillo M.D., Fernandez-Gomez B., Martinez-Saez N., Iriondo-DeHond A., Mesa M.D., Farah A. (2019). Coffee by-products. Coffee: Production, Quality and Chemistry.

[40] EFSA NDA Panel (EFSA Panel on Nutrition, Novel Foods and Food Allergens) (2022). Safety of Dried Coffee Husk (Cascara) from *Coffea arabica* L. as a Novel Food Pursuant to Regulation (EU) 2015/2283. EFSA J..

[41] Iriondo-DeHond M., Iriondo-DeHond A., Herrera T., Fernández-Fernández A.M., Sorzano C.O.S., Miguel E., del Castillo M.D. (2020). Sensory acceptance, appetite control and gastrointestinal tolerance of yogurts containing coffee-cascara extract and inulin. Nutrients.

[42] Einfalt D., Meissner K., Kurz L., Intami K., Müller J. (2020). Fruit spirit production from coffee cherries—Process analysis and sensory evaluation. Beverages.

[43] Blumenthal P., Steger M.C., Bellucci A.Q., Segatz V., Rieke-Zapp J., Sommerfeld K., Schwarz S., Einfalt D., Lachenmeier D.W. (2022). Prodution of coffee cherry spirits from *Coffea arabica* varieties. Foods.

[44] Muzaifa M., Andini R., Sulaiman M.I., Abubakar Y., Rahmi F., Nurzainura (2021). Novel utilization of coffee processing by-products: Kombucha cascara originated from ‘Gayo-Arabica’. IOP Conf. Ser. Earth Environ. Sci..

[45] Huynh-Ba T. (1995). Preparation of Quinic Acid Derivatives. U.S. Patent.

[46] Villarreal-Soto S.A., Bouajila J., Pace M., Leech J., Cotter P.D., Souchard J.-P., Taillandier P., Beaufort S. (2020). Metabolome-microbiome signatures in the fermented beverage, Kombucha. Int. J. Food Microbiol..

[47] Nummer B.A. (2013). Kombucha brewing under the Food and Drug Administration model food code: Risk analysis and processing guidance. J. Environ. Health.

[48] Yamanaka L.E., Abdala P., Christoff A.P. (2018). Extração de DNA: Avaliação da Metodologia Utilizada pela Neoprospecta. Neoprospecta Microbiome Technologies. Nota Técnica: Extração de DNA Neo..

[49] Andrews S. (2010). FastQC: A Quality Control Tool for High Throughput Sequence Data. http://www.bioinformatics.babraham.ac.uk/projects/fastqc.

[50] Smyth R.P., Schlub T.E., Grimm A., Venturi V., Chopra A., Mallal S., Davenport M.P., Mak J. (2010). Reducing chimera formation during PCR amplification to ensure accurate genotyping. Gene.

[51] Altschul S.F., Gish W., Miller W., Myers E.W. (1990). Basic local alignment search tool. J. Mol. Biol..

[52] Instituto Adolfo Lutz (2008). Métodos Físico-Químicos para Análise de Alimentos.

[53] Wischral D., Arias J.M., Modesto L.F., Passos D.F., Pereira N. (2019). Lactic acid production from sugarcane bagasse hydrolysates by *Lactobacillus pentosus*: Integrating xylose and glucose fermentation. Biotechnol. Prog..

[54] Liang H., Liang Y., Dong J., Lu J. (2007). Tea extraction methods in relation to control of epimerization of tea catechins. J. Sci. Food Agric..

[55] Farah A., de Paulis T., Moreira D.P., Trugo L.C., Martin P.R. (2006). Chlorogenic acids and lactones in regular and water-decaffeinated arabica coffees. J. Agric. Food Chem..

[56] Duarte G.S., Farah A. (2011). Effect of simultaneous consumption of milk and coffee on chlorogenic acids’ bioavailability in humans. J. Agric. Food Chem..

[57] Farah A., Monteiro M.C., Calado V., Franca A.S., Trugo L.C. (2006). Correlation between cup quality and chemical attributes of Brazilian coffees. Food Chem..

[58] Bakondi E., Gönczi M., Szabó É., Bai P., Pacher P., Gergely P., Virág L. (2003). Role of intracellular calcium mobilization and cell-density-dependent signaling in oxidative-stress-induced cytotoxicity in HaCaT keratinocytes. J. Investig. Dermatol..

[59] Gomes A., Fernandes E., Lima J.L.F.C. (2005). Fluorescence probes used for detection of reactive oxygen species. J. Biochem. Biophys. Methods.

[60] IriondoDeHond A., Abdolmaleky H.M., Zhou J.R., Del Castillo M.D. (2018). Assesment of antioxidante and anti-inflammatory effects of coffee by-product extracts on a cellular model of diabetic nephropathy. Nutr. Clín. Diet. Hosp..

[61] Benayad Z., Martinez-Villaluenga C., Frias J., Gomez-Cordoves C., Es-Safi N.E. (2014). Phenolic Composition, Antioxidant and Anti-Inflammatory Activities of Extracts from Moroccan Opuntia Ficus-Indica Flowers Obtained by Different Extraction Methods. Ind. Crops Prod..

[62] Bishop P., Pitts E.R., Budner D., Thompson-Witrick K.A. (2022). Kombucha: Biochemical and microbiological impacts on the chemical and flavor profile. Food Chem..

[63] Arikan M., Mitchell A.L., Finn R.D., Gürel F. (2020). Microbial composition of Kombucha determined using amplicon sequencing and shotgun metagenomics. J. Food Sci..

[64] Marsh A., O’Sullivan O., Hill C., Ross R.P., Cotter P.D. (2014). Sequence-based analysis of the bacterial and fungal compositions of multiple kombucha (tea fungus) samples. Food Microbiol..

[65] Landis E.A., Fogarty E., Edwards J.C., Popa O., Eren A.M., Wolfe B.E. (2022). Microbial diversity and interaction specificity in kombucha tea fermentations. mSystems.

[66] Lavasani P.S., Motevaseli E., Sanikhani N.S., Modarressi M.H. (2019). *Komagataeibacter xylinus* as a novel probiotic candidate with high glucose conversion rate properties. Heliyon.

[67] Liu M., Chen Q., Sun Y., Zeng L., Wu H., Gu Q., Li P. (2022). Probiotic potential of a folate-producing strains *Latilactobacillussakei*LZ217 and its modulation effects on human gut Microbiota. Foods.

[68] Qi Y., Huang L., Zeng Y., Li W., Zhou D., Xie J., Xie J., Tu Q., Deng D., Yin J. (2021). *Pediococcus pentosaceus*: Screening and application as probiotic in food processing. Front. Mirobiol..

[69] Singh N., Anand S., Fuquay J.W. (2011). Enterobacteriaceae. Encyclopedia of Dairy Sciences.

[70] Albergaria H., Arneborg N. (2016). Dominance of *Saccharomyces cerevisiae* in alcoholic fermentation processes: Role of physiological fitness and microbial interactions. Appl. Microbiol. Biotechnol..

[71] Staniszewski A., Kordowska-Wiater M. (2021). Probiotic and potentially probiotic yeasts—Characteristics and food application. Foods.

[72] Vejarano R. (2018). *Saccharomycodes ludwigii*, control and potential uses in winemaking process. Fermentation.

[73] Teoh A.L., Heard G., Cox J. (2004). Yeast ecology of Kombucha fermentation. Int. J. Food Microbiol..

[74] Da Silva Júnior J.C., Magnani M., da Costa W.K.A., Madruga M.S., Olegário L.S., Borges G.S.C., Santas A.M., Lima M.S., de Lima L.C., Brito I.L. (2021). Traditional and flavored kombuchas with pitanga and umbu-cajá pulps: Chemical properties, antioxidants, and bioactive compounds. Food Biosci..

[75] May A., Narayanan S., Alcock J., Varsani A., Maley C., Aktips A. (2019). Kombucha: A novel model system for cooperation and conflict in a complex multi-species microbial ecosystem. PeerJ.

[76] Jafari R., Naghavi N.S., Khosravi-Darani K., Doudi M., Shahanipour K. (2020). Kombucha microbial starter with enhanced production of antioxidant compounds and invertase. Biocatal. Agric. Biotechnol..

[77] Jayabalan R., Marimuthu S., Swaminathan K. (2007). Changes in content of organic acids and tea polyphenols during kombucha tea fermentation. Food Chem..

[78] De Noronha M.C., Cardoso R.R., D’Almeida C.T.S., do Carmo M.A.V., Azevedo L., Maltarollo V.G., Ribeiro Júnior J.I., Eller M.R., Cameron L.C., Ferreira M.S.L. (2022). Black tea kombucha: Physicochemical, microbiological and comprehensive phenolic profile changes during fermentation, and antimalarial activity. Food Chem..

[79] Centers for Disease Control and Prevention Get the Facts: Sugar-Sweetened Beverages and Consumption. https://www.cdc.gov/nutrition/data-statistics/sugar-sweetened-beverages-intake.html.

[80] Brazilian Institute of Geography and Statistics (IBGE) (2020). Pesquisa de Orçamentos Familiares 2017–2018: Análise do Consumo Alimentar Pessoal No Brasil, Coordenação de Trabalho e Rendimento.

[81] Eurostat How Often Do You Drink Sugar-Sweetened Soft Drinks?. https://ec.europa.eu/eurostat/web/products-eurostat-news/-/ddn-20210727-1.

[82] Koch W., Kukula-Koch W., Glowniak K. (2017). Catechin composition and antioxidant activity of black teas in relation to brewing time. J. AOAC Int..

[83] Gaggia F., Baffoni L., Galiano M., Nielsen D.S., Jakobsen R.R., Castro-Mejía J.L., Bosi S., Truzzi F., Musumeci F., Dinelli G. (2019). Kombucha beverage from green, black and rooibos teas: A comparative study looking at microbiology, chemistry and antioxidant activity. Nutrients.

[84] Crozier A., Del Rio D., Clifford M.N. (2010). Bioavailability of dietary flavonoids and phenolic compounds. Mol. Aspects Med..

[85] Alberto M.R., Gómez-Cordovés C., de Nadra M.C.M. (2004). Metabolism of gallic acid and catechin by *Lactobacillus hilgardii* from wine. J. Agric. Food Chem..

[86] Farah A., Lima J.P. (2019). Consumption of chlorogenic acids through coffee and health implications. Beverages.

[87] Li C., Lee M.-J., Sheng S., Meng X., Prabhu S., Winnik B., Huang B., Chung J.Y., Yan S., Ho C.-T. (2000). Structural identification of two metabolites of catechins and their kinetics in human urine and blood after tea ingestion. Chem. Res. Toxicol..

[88] Stoikidou T., Koidis A., Zabetakis I., Tsoupras A., Lordan R., Ramji D. (2023). Chapter 2-Coffee and tea bioactive compounds. Functional Foods and Their Implications for Health Promotion.

[89] Farah A., Donangelo C.M. (2006). Phenolic compounds in coffee. Braz. J. Plant. Physiol..

[90] Pereira G.V.M., Carvalho Neto D.P., Magalhães Júnior A.I., do Prado F.G., Pagnoncelli M.G.B., Karp S.G., Soccol C.R., Toldrá F. (2020). Chemical composition and health properties of coffee and coffee by-products. Advances in Food and Nutrition Research.

[91] Plumb G.W., Garcia-Conesa M.T., Kroon P.A., Rhodes M., Ridley S., Williamson G. (1999). Metabolism of chlorogenic acid by human plasma, liver, intestine and gut microflora. J. Sci. Food Agric..

[92] da Silveira J.S., Durand N., Lacour S., Belleville M.-P., Perez A., Loiseau G., Dornier M. (2019). Solid-state fermentation as a sustainable method for coffee pulp treatment and production of an extract rich in chlorogenic acids. Food Bioprod. Process..

[93] Riva A., Kolimár C., Spittler A., Wisgrill L., Herbold C.W., Abrankó L., Berry D. (2020). Conversion of rutin, a prevalent dietary flavonol, by the human gut microbiota. Front. Microbiol..

[94] Ramirez-Martinez J.R. (1988). Phenolic compounds in coffee pulp: Quantitative determination by HPLC. J. Sci. Food Agric..

[95] Mullen W., Nemzer B., Stalmach A., Ali S., Combet E. (2013). Polyphenolic and hydroxycinnamate contents of whole coffee fruits from China, India and Mexico. J. Agric. Food Chem..

[96] Niwa T. (2010). Indoxyl sulfate is a nephro-vascular toxin. J. Renal Nutr..

[97] Cheng T.-H., Ma M.-C., Liao M.-T., Zheng C.M., Lu K.C., Liao C.H., Hou Y.-C., Liu W.-C., Lu C.-L. (2020). Indoxyl sulfate, a tubular toxin, contributes to the development of chronic kidney disease. Toxins.

[98] Ellis R.J., Small D.M., Vesey D.A., Johnson D.W., Francis R., Vitetta L., Gobe G.C., Morais C. (2016). Indoxyl sulfate and kidney disease: Causes, consequences and interventions. Nephrology.

[99] Ellis R.J., Small D.M., Ng K.L., Vesey D.A., Vitetta L., Francis R.S., Gobe G.C., Morais C. (2018). Indoxyl sulfate induces apoptosis and hypertrophy in human kidney proximal tubular cells. Toxicol. Pathol..

[100] Azqueta A., Stopper H., Zegura B., Dusinska M., Moller P. (2022). Do cytotoxicity and cell death cause false positive results in the in vitro comet assay?. Mutat. Res. Genet. Toxicol. Environ. Mutagen..

[101] Rosas-Villegas A., Sánchez-Tapia M., Avila-Nava A., Ramírez V., Tovar A.R., Torres N. (2017). Differential effect of sucrose and fructose in combination with a high fat diet on intestinal microbiota and kidney oxidative stress. Nutrients.

[102] Bhattacharya S., Gachhui R., Sil P.C. (2013). Effect of Kombucha, a fermented black tea in attenuating oxidative stress mediated tissue damage in alloxan induced diabetic rats. Food Chem. Toxicol..

[103] Sun X.-Y., Wang J.M., Ouyang J.-M., Kuang L. (2018). Antioxidant activities and repair effects on oxidatively damaged HK-2 cells of tea polysaccharides with different molecular weights. Oxid. Med. Cell. Longev..

[104] Visioli F., De La Lastra C.A., Andres-Lacueva C., Aviram M., Calhau C., Cassano A., D’Archivo M., Faria A., Favé G., Fogliano V. (2011). Polyphenols and human health: A prospectus. Crit. Rev. Food Sci. Nutr..

[105] Shen N., Wang T., Gan Q., Liu S., Wang L., Jin B. (2022). Plant flavonoids: Classification, distribution, biosynthesis, and antioxidant activity. Food Chem..

[106] Hussain T., Tan B., Yin Y., Blachier F., Tossou M.C.B., Rahu N. (2016). Oxidative stress and inflammation: What polyphenols can do for us?. Oxid. Med. Cell. Longev..

[107] Bernatoniene J., Kopustinskiene D.M. (2018). The role of catechins in cellular responses to oxidative stress. Molecules.

[108] Rebollo-Hernanz M., Zhang Q., Aguilera Y., Martín-Cabrejas M.A., de Mejia E.G. (2019). Relationship of the phytochemicals from coffee and cocoa by-products with their potential to modulate biomarkers of metabolic syndrome in vitro. Antioxidants.

[109] Rebollo-Hernanz M., Zhang Q., Aguilera Y., Martín-Cabrejas M.A., de Mejia E.G. (2019). Phenolic compounds from coffee by-products modulate adipogenesis-related inflammation, mitochondrial dysfunction, and insulin resistance in adipocytes, via insulin/PI3K/AKT signaling pathways. Food Chem. Toxicol..

[110] Ludwig I.A., Clifford M.N., Lean M.E.J., Ashihara H., Crozier A. (2014). Coffee: Biochemistry and potential impact on health. Food Funct..

[111] Lima J.P., Farah A., Farah A. (2019). Caffeine metabolism and health effects. Coffee: Consumption and Health Implications.

[112] Chaudhary K., Malhota K., Sowers J., Arror A. (2013). Uric Acid—Key ingredient in the recipe for cardiorenal metabolic syndrome. Cardiorenal Med..

[113] Kocak M.Z., Aktas G., Duman T.T., Atak B.M., Savli H. (2019). Is Uric Acid elevation a random finding or a causative agent of diabetic nephropathy?. Rev. Assoc. Med. Bras..

[114] Khan N.U., Lin J., Liu X., Li H., Lu W., Zong Z., Zhang H., Waqas M., Shen L. (2020). Insights into predicting diabetic nephropathy using urinary biomarkers. Biochim. Biophys. Acta Proteins Proteom..

[115] Mahmoodpoor F., Saadat Y.R., Barzegari A., Ardalan M., Vahed S.Z. (2017). The impact of gut microbiota on kidney function and pathogenesis. Biomed. Pharmacother..

[116] Hou C., Liu D., Wang M., Gong C., Li Y., Yang L., Yao M., Yuan E., Ren J. (2019). Novel xanthine oxidase-based cell model using HK-2 cell for screening antihyperuricemic functional compounds. Free. Radic. Biol. Med..

[117] Zhu H., Song D., Zhao X. (2021). Potential applications and preliminary mechanism of action of dietary polyphenols against hyperuricemia: A review. Food Biosci..

[118] Zhou X., Zhang B., Zhao X., Lin Y., Wang J., Wang X., Hu N., Wang S. (2021). Chlorogenic acid supplementation ameliorates hyperuricemia, relieves renal inflammation, and modulates intestinal homeostasis. Food Funct..

[119] Liu A., Jiang C., Liu Q., Yn H., Zhou H., Ma H., Geng Q. (2022). The inverted U-Shaped association of caffeine intake with serum uric acid in U.S. adults. J. Nutr. Health Aging.

[120] Zhu L.-Q., Fan X.H., Li J.-F., Chen J.-H., Liang Y., Hu X.L., Ma S.M., Hao X.-Y., Shi T., Wang Z. (2021). Discovery of a novel inhibitor of nitric oxide production with potential therapeutic effect on acute inflammation. Bioorg. Med. Chem. Lett..

[121] Moghaddam A.S., Mohammadian S., Vazini H., Taghadosi M., Esmaeili S.A., Mardani F., Seifi B., Mohammadi A., Afshari J.T., Sahebkar A. (2018). Macrophage plasticity, polarization and function in health and disease. J. Cell. Physiol..

[122] Wynn T.A., Chawla A., Pollard J.W. (2013). Macrophage biology in development, homeostasis and disease. Nature.

[123] Lim A.K.H., Tesch G.H. (2014). Inflammation in diabetic nephropathy. World J. Diabetes.

[124] Wu Z., Huang Y., Hu W., Ren L., Jiang P., Margolskee R.F., Wang H., Feng S. (2022). Lipopolysaccharide-induced inflammation increases nitric oxide production in taste buds. Brain Behav. Immun..

[125] Page M.J., Kell D.B., Preterius E. (2022). The role of lipopolysaccharide-induces cell signalling in chronic inflammation. Chronic Stress..

[126] Sharma J.N., Al-Omran A., Parvathy S.S. (2007). Role of nitric oxide in inflammatory diseases. Inflammopharmacology.

[127] Lin Y.-S., Wu S.-S., Lin J.-K. (2003). Determination of tea polyphenols and caffeine in tea flowers (*Camellia sinensis*) and their hydroxyl radical scavenging and nitric oxide suppressing effects. J. Agric. Food Chem..

[128] Novilla A., Djamhuri D.S., Nurhayati B., Rihibiha D.D., Afifah E., Widowati W. (2017). Anti-inflammatory properties of oolong tea (*Camellia sinensis*) ethanol extract and epigallocatechin gallate in LPS-induced RAW 264.7 cells. Asian Pac. J. Trop. Biomed..

[129] Wang P., Feng Z., Sang X., Chen W., Zhang X., Xiao J., Chen Y., Chen Q., Yang M., Du J. (2021). Kombucha ameliorates LPS-induced sepsis in a mouse model. Food Funct..

[130] Vázquez-Cabral B.D., Larrosa-Pérez M., Gallegos-Infante J.A., Moreno-Jiménez M.R., González-Laredo R.F., Rutiaga-Quiñones J.G., Gamboa-Gómez C.I., Rocha-Guzmán N.E. (2017). Oak kombucha protects against oxidative stress and inflammatory processes. Chem.-Biol. Interact..

[131] Tang J., Diao P., Shu X., Li L., Xiong L. (2019). Quercetin and quercitrin attenuates the inflammatory response and oxidative stress in LPS-induced RAW 264.7 cells: In vitro assessment and a theoretical model. Biomed. Res. Int..

[132] Lin H.-Y., Juan S.-H., Shen S.-C., Hsu F.L., Chen Y.-C. (2003). Inhibition of lipopolysaccharide-induced nitric oxide production by flavonoids in RAW 264.7 macrophages involves heme oxygenase-1. Biochem. Pharmacol..

[133] Xu J., Zhao Y., Aisa H.A. (2017). Anti-inflammatory effect of pomegranate flower in lipopolysaccharide (LPS)-stimulated RAW 264.7 macrophages. Pharm. Biol..

[134] Gu I., Brownmiller C., Stebbind N.B., Mauromoustakos A., Howard L., Lee S.-O. (2020). Berry phenolic and volatile extracts inhibit pro-inflammatory cytokine secretion in LPS—Stimulated RAW 264.7 cells throught suppression of NK-kB signaling pathway. Antioxidants.

[135] Fernández-Fernández A.M., Dellacassa E., Nardin T., Larcher R., Gámbaro A., Medrano-Fernandez A., del Castillo M.D. (2021). In vitro bioaccessibility of bioactive compounds from citrus pomaces and orange pomace biscuits. Molecules.

[136] Gutierrez-Barrutia M.B., Cozzano S., Arcia P., del Castillo M.D. (2022). In vitro digestibility and bioaccessibility of nutrients and non-nutrients composing extruded brewers’ spent grain. Nutrients.

[137] Hwang J.H., Kim K.J., Ryu S.J., Lee B.-Y. (2016). Caffeine prevents LPS-induced inflammatory responses in RAW 264.7 cells and zebrafish. Chem. -Biol. Interact..

