# Supplementary material for: Intracellular Antioxidant and Anti-Inflammatory Effects and Bioactive Profiles of Coffee Cascara and Black Tea Kombucha Beverages
Source: Foods. 2023 May 6;12(9):1905. doi: 10.3390/foods12091905 (PMC10177953; doi:10.3390/foods12091905)
Supplement: Supplementary file 1 [file foods-12-01905-s001.zip › foods-2327865-supplementary.pdf]

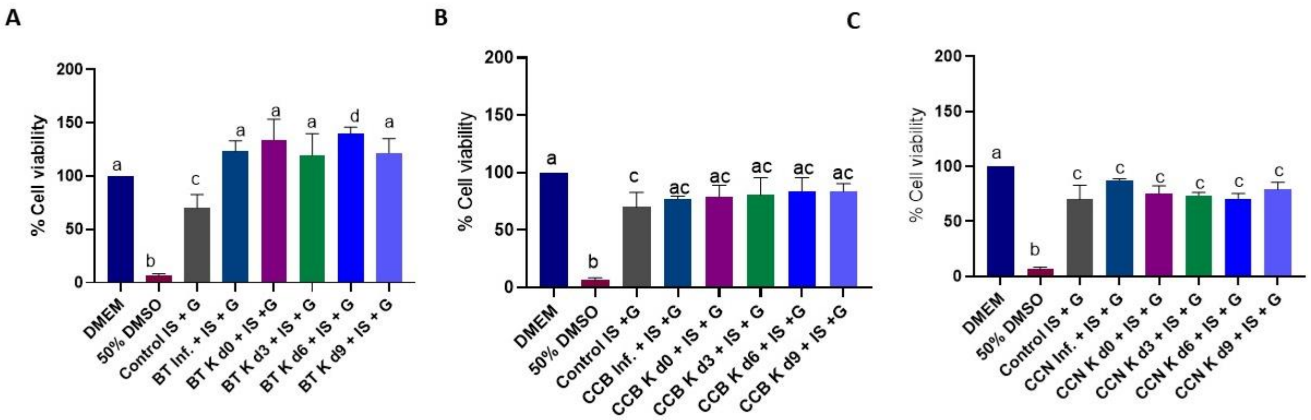

Figure S1. Effect of treatment with BT (A), CCB (B) and CCN (C) beverages, associated with IS and high G, on HK-2 cells viability, determined by MTT assay. Results are average of triplicate of experiments performed three times. Different letters over the bars indicate statistical difference among treatments by ANOVA followed by Tukey's test ($p < 0.05$). DMEM: Dulbecco's Modified Eagle Media (life control); DMSO: Dimethyl Sulphoxide (death control). IS: indoxyl sulfate; G: culture media with high glucose solution; d0, d3, d6 and d9: days 0, 3, 6 and 9 of fermentation, respectively; BT: black tea; Inf: plain infusion (no starter and sugar); K: kombucha; CCB: coffee cascara from Brazil; CCN: coffee cascara from Nicaragua. . Different letters over the bars indicate statistical difference among treatments by ANOVA followed by Tukey's test ($p < 0.05$).

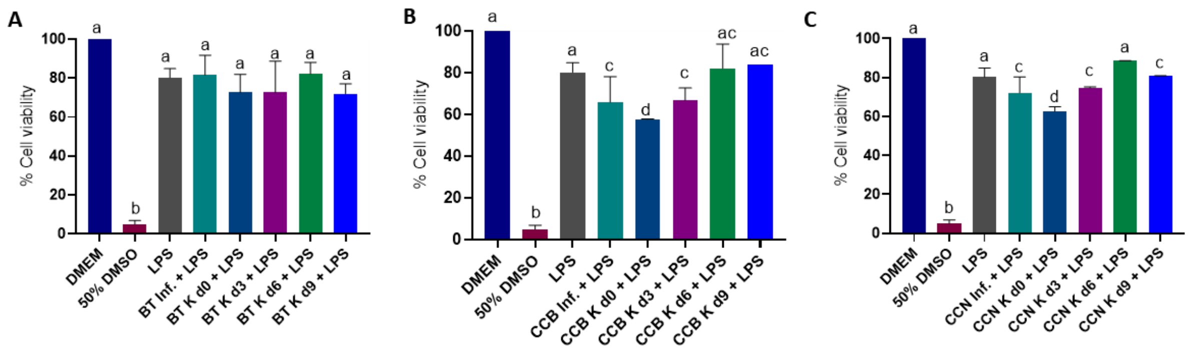

Figure S2. Effect of treatment with BT (A), CCB (B) or CCN (C) beverages, associated with LPS, on RAW 264.7 cells viability, determined by MTT assay. Results are mean of triplicate of experiments performed three times. DMEM: Dulbecco's Modified Eagle Media (life control); DMSO: Dimethyl Sulphoxide (death control). d0, d3, d6 and d9: days 0, 3, 6 and 9 of fermentation, respectively.BT: black tea; Inf: infusion; K: kombucha; CCB: Coffee cascara from Brazil; CCN: Coffee cascara from Nicaragua. Different letters over the bars indicate statistical difference among treatments by ANOVA followed by Tukey's test ($p < 0.05$).
